# Comprehensive Structural and Molecular Comparison of Spike Proteins of SARS-CoV-2, SARS-CoV and MERS-CoV, and Their Interactions with ACE2

**DOI:** 10.3390/cells9122638

**Published:** 2020-12-08

**Authors:** Ma’mon M. Hatmal, Walhan Alshaer, Mohammad A. I. Al-Hatamleh, Malik Hatmal, Othman Smadi, Mutasem O. Taha, Ayman J. Oweida, Jennifer C. Boer, Rohimah Mohamud, Magdalena Plebanski

**Affiliations:** 1 Department of Medical Laboratory Sciences, Faculty of Applied Health Sciences, The Hashemite University, Zarqa 13133, Jordan; 2 Cell Therapy Center (CTC), The University of Jordan, Amman 11942, Jordan; 3 Department of Immunology, School of Medical Sciences, Universiti Sains Malaysia, Kubang Kerian, Kelantan 16150, Malaysia; alhatamleh@student.usm.my (M.A.I.A.-H.); rohimahm@usm.my (R.M.); 4 Prince Hamza Hospital, Amman 11947, Jordan; hatmal.malik@yahoo.com; 5 Department of Biomedical Engineering, Faculty of Engineering, The Hashemite University, Zarqa 13133, Jordan; othman.smadi@hu.edu.jo; 6 Drug Design and Discovery Unit, Department of Pharmaceutical Sciences, Faculty of Pharmacy, The University of Jordan, Amman 11942, Jordan; mutasem@ju.edu.jo; 7 Department of Nuclear Medicine and Radiobiology, Faculty of Medicine and Health Sciences, Université de Sherbrooke, Sherbrooke, QC J1H 5N4, Canada; ayman.oweida@usherbrooke.ca; 8 Translational Immunology and Nanotechnology Unit, School of Health and Biomedical Sciences, RMIT University, Bundoora 3083, Australia; jennifer.boer@rmit.edu.au (J.C.B.); magdalena.plebanski@rmit.edu.au (M.P.); 9 Hospital Universiti Sains Malaysia, Health Campus, Kubang Kerian, Kelantan 16150, Malaysia

**Keywords:** COVID-19, coronaviruses, spike protein, receptor-binding motif, terminal region, proline

## Abstract

The severe acute respiratory syndrome coronavirus-2 (SARS-CoV-2) has recently emerged in China and caused a disease called coronavirus disease 2019 (COVID-19). The virus quickly spread around the world, causing a sustained global outbreak. Although SARS-CoV-2, and other coronaviruses, SARS-CoV and Middle East respiratory syndrome CoV (MERS-CoV) are highly similar genetically and at the protein production level, there are significant differences between them. Research has shown that the structural spike (S) protein plays an important role in the evolution and transmission of SARS-CoV-2. So far, studies have shown that various genes encoding primarily for elements of S protein undergo frequent mutation. We have performed an in-depth review of the literature covering the structural and mutational aspects of S protein in the context of SARS-CoV-2, and compared them with those of SARS-CoV and MERS-CoV. Our analytical approach consisted in an initial genome and transcriptome analysis, followed by primary, secondary and tertiary protein structure analysis. Additionally, we investigated the potential effects of these differences on the S protein binding and interactions to angiotensin-converting enzyme 2 (ACE2), and we established, after extensive analysis of previous research articles, that SARS-CoV-2 and SARS-CoV use different ends/regions in S protein receptor-binding motif (RBM) and different types of interactions for their chief binding with ACE2. These differences may have significant implications on pathogenesis, entry and ability to infect intermediate hosts for these coronaviruses. This review comprehensively addresses in detail the variations in S protein, its receptor-binding characteristics and detailed structural interactions, the process of cleavage involved in priming, as well as other differences between coronaviruses.

## 1. Introduction

A novel coronavirus (severe acute respiratory syndrome coronavirus-2, or SARS-CoV-2) emerged in China in 2019. It causes illness in humans termed coronavirus disease 2019 (COVID-19). The symptoms of this disease are similar to those of SARS-CoV in humans, and can lead to fatal respiratory tract infection. Phylogenetic studies have shown that SARS-CoV-2 is a beta-coronavirus [1] that belongs to a group of SARS-CoV and SARS-like bat-derived viruses (ZC45 and ZCX21) [2]. Coronaviruses are composed of short-strand ribonucleic acid (ssRNA) as genetic material. The RNA strands in coronaviruses range in length from 26 to 32 kbs [3].

In the early weeks of the COVID-19 pandemic, the WHO expected that the production of an effective vaccine against SARS-CoV-2 would probably require 18 months of development. This encouraged researchers to assess the potential antiviral effects of existing drugs against COVID-19, as such, an approach could be cost- and time-effective amid the current situation [4]. Extensive trials have been performed to investigate the possibility of repurposing currently-used FDA approved drugs to treat COVID-19. There are also many drugs in development to target the virus directly. For example, a protease inhibitor, camostat mesylate, has been demonstrated to inhibit SARS-CoV-2 infection and prevent the entry of the virus mediated by the spike (S) protein into primary human lung cells [5,6]. The S proteins offer a good druggable target, since they cover the virus surface, and are key to initiate binding to the host cell receptors angiotensin converting enzyme 2 (ACE2) and the transmembrane serine protease (TMPRSS2), leading to subsequent internalization and infection.

ACE2 is a type I membrane zinc-containing enzyme that is expressed on the surface of endothelial cells in the lungs, as well as some other cell types and tissues [7]. Specifically, ACE2 is attached to the cell membrane of main pulmonary type II cells, the entericytes of the small intestines, arterial and vein endothelial cells and smooth arterial muscle cells in most organs. The protein of ACE2 contains the N-terminal peptidase M2 domain and the C-terminal amino acid collectrin transporter domain [8].

The amino acids that interact at the ACE2 and SARS-CoV-2 receptor-binding domain (RBD) interface have been analyzed through the crystal structure (PDB IDs: 6LZG and 6M17) [2,9,10]. In total, 15 residues from ACE2 interact with the RBD: residues Gln24, Thr27, Asp30, Lys31, His34, Glu35, Glu37, Asp38, Tyr41 and Gln42 are in α1, one residue (residue Met82) comes from α2, while the residues Lys353, Gly354, Asp355 and Arg357 come from the linker between β3 and β4. Therefore, these 15 amino acids in ACE2 can be labelled as critical amino acids for binding, and the α1, α2, β3, and β4 secondary structure elements as ACE2 critical binding components to the S protein [9].

Many studies have been performed to design a therapeutic peptide based on ACE2, which could act as an antagonist for binding. In one study, it was found that the single α1-helix used in inhibitor 1 was somewhat unstable, whereas the α1 and α2-helices that were used in inhibitors 2–4 supported each other and retained their bent shape, which provided a conformational match to the RBD of SARS-CoV-2, and fully covered the RBD surface. Precise conformational matching between the designed peptides and the virus provides an opportunity to further improve the binding affinity, which should be considered in future inhibitor design protocols [9]. Moreover, Battle and associates also proposed the use of the already available recombinant ACE2 to intercept COVID-19 and, therefore, to attenuate infection [11,12].

Studies have shown that proteins of different coronaviruses display sequence variations that cause differences in the three dimensional (3D) structures, and, thus, in the shape of their binding contacts [13]. The current review compares the structure of S protein from SARS-CoV-2, SARS-CoV and MERS-CoV, and shows the genetic variability between them, with pinpointing potentially significant variations capable of altering binding contacts. Understanding the potential effects of these variations would be helpful towards determining the range of available intermediate hosts, as well as their effects on immunogenicity and treatment. Structural information at this atomic level has the potential to improve our understanding of the interactions between SARS-CoV-2 and its variants with infection susceptible cells, help identify robust target regions for neutralizing antibodies and assist with structure-based drug and vaccine design, urgently needed in the fight against SARS-CoV-2 [6].

## 2. Genome Comparison of Coronaviruses

The S protein amino acids variations among different coronaviruses and ACE2 differences suggest which species can be natural reservoirs of SARS-CoV-2. Coronaviruses carry the largest genomes of all those found in RNA virus families, and they possess a 30 nucleotide polyadenosine (A) tail [14]. The SARS-CoV-2 virus shares 80% of its genome with the other human coronaviruses [3]. The bat coronavirus RatG13 appears to be the closest relative of SARS-CoV-2, as they share over a 93.1% sequence identity in the S gene [3] and a 98% sequence identity in the S protein [15]. Bat CoV ZXC21 and CoV ZC45 also bear very close genome similarity (87.99% and 87.23% sequence identity, respectively) with SARS-CoV-2, while the S gene exhibited only around 75% sequence identity [16].

**Figure 1:**
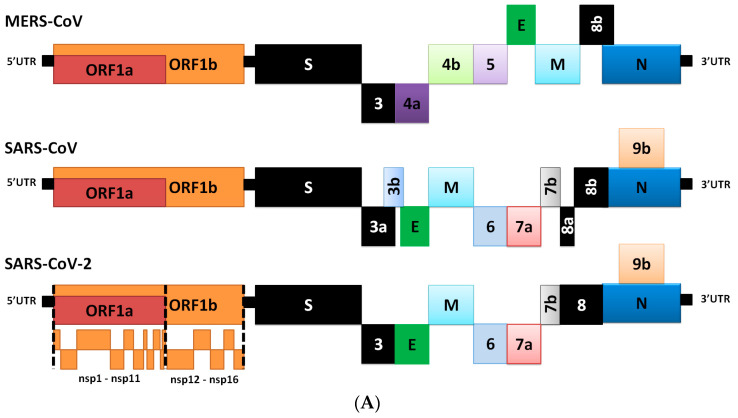

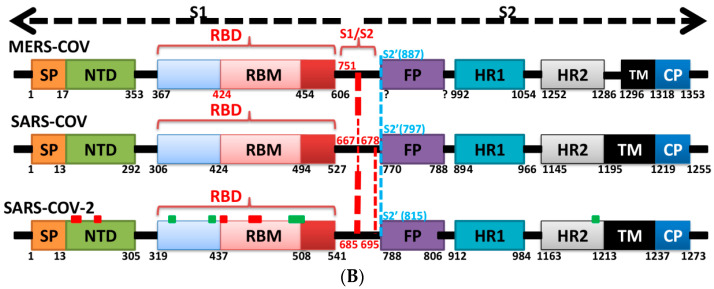
The general structures of Middle East respiratory syndrome CoV (MERS-CoV), severe acute respiratory syndrome coronavirus (SARS-CoV) and SARS-CoV-2 genome and S protein. (**A**) The gene structure of MERS-CoV, SARS-CoV and SARS-CoV-2. Black boxes represent the most critical differences between the viruses. The open reading frames (ORF)1a produces polypeptide 1a (pp1a, 440–500 kDa), which is cleaved into 11 nsps. A frameshift occurs immediately upstream of the ORF1a stop codon, which allows continued translation of ORF1ab to yield a large polypeptide (pp1ab, 740–810 kDa), which is cleaved into 15 nsps. The proteolytic cleavage is mediated by viral proteases nsp3 and nsp5, which harbor a papain-like protease domain and a 3C-like protease (3CL^pro^), respectively. The viral genome is also used as the template for replication and transcription, which is mediated by nsp12, which harbors RNA-dependent RNA polymerase (RdRp) activity [14,18,19]. (**B**) The structure of S protein from of MERS-CoV, SARS-CoV and SARS-CoV-2. S1/S2: the potential furin cleavage sites between S1 and S2 domains of S protein; S2′: S2′ protease furin cleavage site; FP: fusion peptide; HR1: heptad repeat 1; HR2: heptad repeat 2 (which is reported to be very flexible in pre-fusion conformation); TM: transmembrane domain; CP: cytoplasmic domain fusion [15]. Vertical dotted lines indicate protease cleavage sites (red for S1/S2 furin-like cleavage sites and blue for S2′ furin-like cleavage site), different line widths represent different propensities for cleavage. MERS-CoV does not contain the second S1/S2 cleavage site. Small red and green boxes in SARS-CoV-2 represent the least and most conservative regions, respectively, in S protein in general and in the receptor-binding domain (RBD) only based on the PAM250 matrix [20] for regions composed of 10 amino acids [15,21].

There are 14 open reading frames (ORFs) that encode for 27 proteins in the SARS-CoV-2 genome. The genes ORF1ab and ORF1a are located at the 5′-end of the SARS-CoV-2 genome. ORF1ab is the largest gene. It encodes the polypeptide (pp) 1ab protein and 15 non-structural proteins (nsps) (nsp1–10 and nsp12–16), while the ORF1a gene encodes pp1a protein, which contains 11 nsps (nsp1–11). Although the evolutionary tree indicates that SARS-CoV-2 is very similar to the rest of the SARS group, recent research has shown remarkable variations between them [3,17]. The main differences are indicated by the black boxes in Figure 1A.

The SARS-CoV-2 genome codes for four structural proteins (spike (S), envelope (E), membrane (M), and nucleocapsid (N)), and eight accessory proteins (3a, 3b, p6, 7a, 7b, 8b, 9b and ORF14) at the 3′-end. The SARS-CoV-2 virus is very similar to SARS-CoV at the amino-acid level, but there are some significant differences [17]. For example, the 8a protein is present in SARS-CoV and absent in SARS-COV-2; the 8b protein is 84 amino acids (aa) in SARS-CoV, but longer in SARS-CoV-2 (121 aa); the 3b protein is 154 aa in SARS-CoV, but is shorter than that in SARS-CoV-2 by 22 aa [17]. Changes have also been detected in the conserved S protein areas [17], which are thought to play significant roles in the virus’ binding affinity [3,17]. Further studies are needed in order to characterize the ways in which these differences affect the functionality and pathogenesis of SARS-CoV-2 [17]. The proteins of SARS-CoV-2 and their functions are described in Table 1 [15]. Amino acids variations in S protein of SARS-CoV-2 (either in the receptor-binding motif (RBM) or other regions) could affect the conformational flexibility of the protein, or binding interactions with ACE2. These effects could be investigated using in-vitro assays (i.e., X-ray crystallography) or using modeling and molecular dynamics approaches [22].

## 3. Differences in Transcriptome

Each coronaviral RNA contains a standard 50 nt lead sequence that is fused from the downstream genome of the body sequence [19]. The coronavirus genome is used as the template for replication and transcription mediated by nsp12 (harboring RNA dependent RNA polymerase (RdRp) activity). Negative-sense RNA intermediates are generated to serve as the templates for the synthesis of positive-sense genomic RNA (gRNA) and subgenomic RNAs (sgRNAs) [14]. The gRNA is packaged by the structural proteins to produce new progeny virions. Shorter sgRNAs encode conserved structural proteins (including S, E, M, N and accessory proteins). SARS-CoV-2 has at least six accessory proteins (3a, 6, 7a, 7b, 8 and 10). However, SARS-CoV-2 ORFs have not yet been experimentally verified for expression. Therefore, it is currently unclear which accessory genes are actually expressed from this compact genome [14].

During negative-strand synthesis, RdRp pauses when it crosses a transcription regulatory sequence (TRS) in the body (TRS-B) and switches to the TRS in the leader (TRS-L), which results in discontinuous transcription leading to the leader-body fusion. This pause results in discontinuous transcription that results in leader-body fusion during negative-strand synthesis. Positive-strand mRNAs are transcribed from the fused negative-strand intermediates. These components can be used to develop diagnostic and therapeutic tools based on the SARS-CoV-2 genome organization. It is unclear whether the general mechanism is applicable to SARS-CoV-2. ORFs that have been deleted or frameshifted may produce short proteins that are distinct from known viral proteins. In addition to the 10 canonical RNA (one genomic and nine subgenomic), SARS-CoV-2 produces transcripts encoding unknown ORFs with fusion, deletion and/or frameshift. In the future, it will be interesting to examine whether these unknown ORFs are translated and whether they produce functional products [14].

The tail of SARS-CoV-2 RNAs has a median length of 47 nt. The full-length gRNA has a longer tail than do sub-genomic RNAs (sgRNAs). SgRNAs have, in particular, two tail populations: a minor of 30 nt and a major of 45 nt [14]. It has already been noted that poly(A) tail lengths change from 45 nt immediately after infection to 65 nt after six to nine hours of infection and to 30 nt after 140 h of infection [24]. It has been shown that nsp8 has an adenylyltransferase activity, which may extend the poly(A) tail of viral RNA [25,26]. The poly(A) tail of mRNA is also critical to control stability and translation through its interaction with poly(A) binding proteins (PABPs). The tail is likely to play multiple roles in translation and replication.

A minimum of 41 sites have been reported to display substantial differences in the transcripts of SARS-CoV-2. Such variety indicates potential RNA modifications. The motif that has been most commonly reported among these modification sites is AAGAA. Change sites that form an “AAGAA-like” pattern (including A/G rich sequences) are especially enriched in 28.5 to 29.5 kb genomic positions. Long viral transcripts (gRNA, S, 3a, E and M) are more frequently modified than short RNAs (6, 7a, 7b, 8 and N), which suggests a modification mechanism that is specific to certain RNA species. Interestingly, modified RNA molecules have shorter poly(A) tails than unmodified ones, which suggests that there is a link between the internal modification and 3′-end tail, and, thus, with viral RNA stability control. It is also plausible that RNA modification is a mechanism to evade host-immune response [14]. It will be interesting, in the future, to identify the chemical nature, enzymology and biological functions of these modifications. The new ORFs may serve as a source of accessory proteins that modulate viral replication and host-immune response. The RNA modifications may also contribute to viral survival and evasion of immunity in infected tissues, as the innate immune system is less sensitive to RNAs with nucleoside modification than to those without modification [14].

## 4. Role of S Protein in Pathogenesis

**Figure 2:**
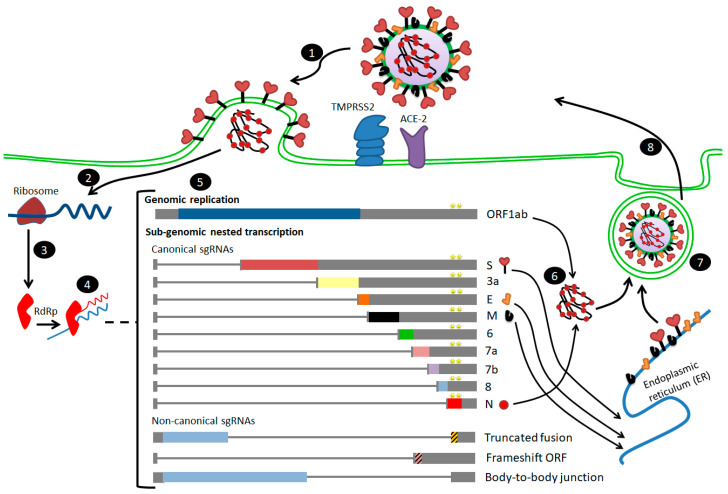
Steps of SARS-CoV-2 infection and entry to the host cell [14,29]. (**1**) After receptor (angiotensin-converting enzyme 2 (ACE2)) binding, the virus enters host cell cytosol via cleavage of S protein by a protease enzyme (transmembrane protease serine 2 (TMPRSS2)), followed by fusion of the viral and cellular membranes. (**2**) Upon cell entry, the genomic RNA is translated to produce non-structural proteins from two open reading frames, ORF1a and ORF1b. (**3**) Some of the nsps have RNA-dependent RNA polymerase (RdRp) activity (nsp12). (**4**) Negative-sense RNA intermediates are generated to serve as the templates for the synthesis of positive-sense genomic RNA (gRNA), and (**5**) sub-genomic RNAs (sgRNAs). (**6**) The gRNA is packaged by the structural proteins to assemble progeny virions. Shorter sgRNAs encode conserved structural proteins (S, E, M and N), and several accessory proteins. SARS-CoV-2 is known to have at least six accessory proteins (3a, 6, 7a, 7b, 8 and 10), and non-canonical sgRNAs are also shown in the figure. The AAGAA-type modification clusters in gRNA and sgRNAs are shown with yellow stars annotations. (**7**,**8**) budding and exocytosis of the virus occur.

The outer surface of the coronavirus SARS-CoV-2 is made up of four structural proteins: S, E, M and N (Table 1 and Figure 1A) [17]. The S protein (~1200 aa long, about 180 kDa) [27] mediates the interaction of the virus with the host cell-surface receptors and their subsequent fusion. Coronavirus replication begins with attachment and entrance of the virus into the host cell, through interactions between the S protein and its receptor. After the receptor attachment to ACE2, the host-cell cytosol enters the virus by the cleavage of S protein (cleavage of S1 from S2) by a protease enzyme (i.e., transmembrane protease serine 2 (TMPRSS2)). This procedure is followed by fusion of the virus and the host-cell membranes. The next step is the translation of the replicase enzyme from the virion genomic RNA, and the translation and assembly of the viral replicase complexes. Encapsidation results in the formation of the mature virus after replication and the subgenomic RNA synthesis. Virions are then transported in vesicles to the cell surface and released through exocytosis [28] (Figure 2). Cryo-electron microscopy studies of the SARS-CoV S protein and its interaction with the cell receptor ACE2 have demonstrated that S1 and ACE2 binding prompts the S2 section to move from a metastable pre-fusion state, to a more stable post-fusion state, which is essential for membrane fusion [6].

Several mutated amino-acid residues in the HR1 domain may be associated with enhanced interactions with the HR2 domain and, thus, enhanced membrane fusion [21]. The presence of a furin-like cleavage site in SARS-CoV-2 has also been found to facilitate the priming of the S protein, and to improve the efficiency of the spread of SARS-CoV-2 compared with other beta coronaviruses [30]. Plasmin and other proteases have been shown to cleave a newly inserted furin site extracellularly in the S protein of SARS-CoV 2, and this procedure increases the virus’ infectivity and virulence. The plasmin system may offer a promising therapeutic target to combat SARS-CoV-2 infection [31]. Collectively, these results indicate SARS-CoV-2 is closely related to SARS-CoV, and suggest SARS-CoV-2 may use various novel glycosylation sites, which may contribute to its pandemic spread, owing to its antigenic discrepancy to previous coronaviruses [32].

## 5. The Primary, Secondary and Tertiary Structures of SARS-CoV-2 S Protein, and Differences from Other Coronaviruses

S protein represents one of the main important viral proteins that should be studied by genotyping of virus isolates, in order to understand the evolution and transmission of SARS-CoV-2. Multiple alignments of sequences of complete genome for SARS-CoV-2 were performed, while the genotyping analysis of SARS-CoV-2 isolates from different places in the world has revealed that the specific multiple mutations are the predominated mutation during the current pandemic [33]. Genotyping analysis has shown that the genes that encode for the S proteins and RNA polymerase, RNA primase and nucleoprotein undergo frequent mutations. Knowledge of these mutations is critical for vaccine development to control the disease [33]. In the S protein of SARS-CoV-2, as well as in other coronaviruses, various amino acid and domain differences have been determined. Figure 1B shows the general domains of S protein and their relevant numbers for MERS-CoV, SARS-CoV and SARS-CoV-2 [15,21,34,35].

The S protein S1 domain mediates receptor attachment. It can be divided into two sub-domains: the N-terminal sub-domain (NTD), which often binds sialic acid; and the C-terminal sub-domain (also known as the C-domain or the receptor-binding domain (RBD)) [36,37]. The fusion peptide is activated through proteolytic cleavage at a site found immediately upstream of these domains (S2′). This pattern is common to all coronaviruses. In many coronaviruses, additional proteolytic priming occurs at a second site that is located at the interface of the S1 and S2 domains (S1/S2) [36,37].

Based on comparison with the S sequence of SARS-CoV, the S1 domain of SARS-CoV-2 has been found to be less preserved (61% identity) than the S2 fusion domain (90% identity). Within S1, the NTD has been found to be less conserved (51% identity) than the RBD (74% identity), which is part of the C-terminal domain (Figure 1B). The level of sequence identity in the RBD is consistent with the view that ACE2 may be used as the host-cell receptor by SARS-CoV-2, as it is by SARS-CoV [1,38,39]. Interestingly, when analyzing the RBM, the identity between the two sequences falls to 50%. In this case, these findings indicate that there are possible differences between the binding residues that are involved in the interactions with the receptor, which, thus, affect binding affinities [1,15,40]. In the well-preserved S2 domain, the identical nature of this domain was high for most sub-regions: the fusion-peptide (FP, 93% identity), heptad-repeat 1 (HR1, 88% identity), heptad-repeat 2 (HR2, 100% identity), transmembrane (TM, 93% identity) and the C-terminal endo domain (E, 97% identity). The SARS-CoV-2 and SARS-CoV FPs, in particular, show high similarity (93% identity) [36], and display the characteristics of viral fusion peptides [27] (Figure 1B).

**Figure 3:**
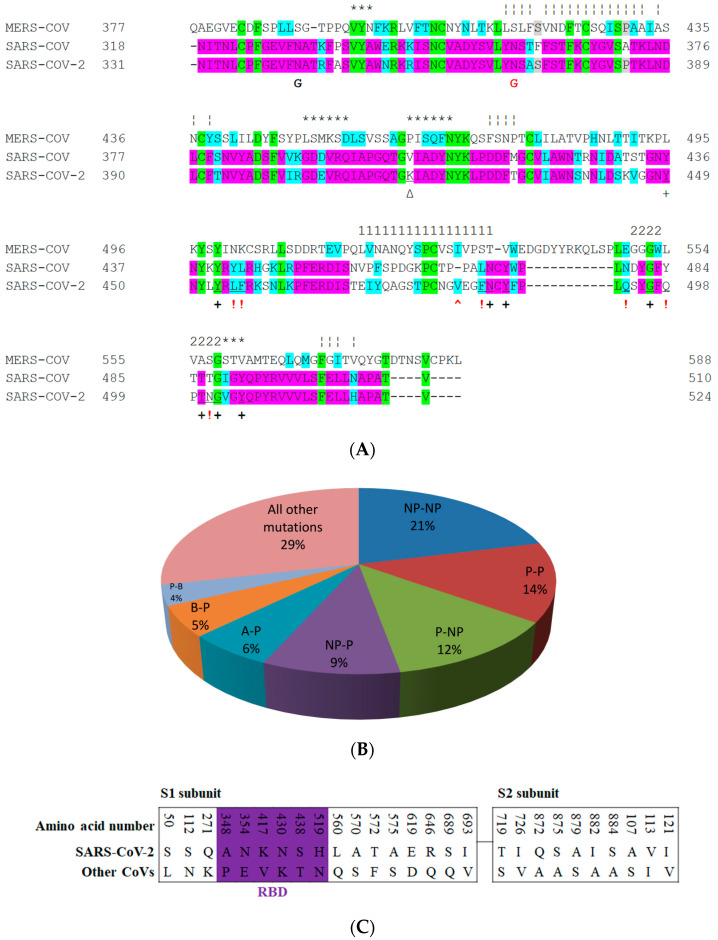
Aligned sequences of RBD of SARS-CoV-2 with other coronaviruses, and rates of all reported mutations in the S protein of SARS-CoV and SARS-CoV-2. (**A**) Aligned sequences (found through use of Clustalw at www.genome.jp/tools-bin/clustalw; [43]) of RBD of SARS-CoV-2 with those of SARS-CoV and MERS-CoV. Green color indicates positions that have a fully conserved residue between the three sequences. Pink color indicates conserved residues between SARS-CoV-2 and SARS-CoV, while grey color indicates conserved residues between SARS-CoV-2 and MERS-CoV. Cyan indicates conservation between residues of strongly similar properties that score > 0.5 in the Gonnet PAM250 matrix [36]. “+” indicates critical, identical amino acids in SARS-CoV-2 and SARS-CoV based on previous studies, while “!” indicates critical, non-identical amino acids in SARS-CoV-2 and SARS-CoV. “Δ” refers to the amino acid that has salt bridges in SARS-CoV-2 but no interactions in SARS-CoV; this amino acid is located outside the RBM motif. “^” refers to the only insertion in the RBD domain (terminal region (TR)1 region) of SARS-CoV-2. “*” indicates amino acids of the upper core region, “1” for TR1, “2” for TR2. “¦” refers to the binding site of the CR3022 antibody, one of the main binding antibodies. “G” refers to potential glycosylated sites (black for conservative glycosylation between SARS-CoV and SARS-CoV-2, while red points to potential glycosylation site only in SARS-CoV). (**B**) Rates of all reported mutations in S protein (284 mutations out of 1255 aa) between SARS-CoV-2 and SARS-CoV for different groups of amino acids (NP: non-polar, non-aromatic. P: polar. Ar: aromatic. A: acidic. B: basic amino acids. Aromatic mutations are included in all other mutations). (**C**) Recorded S protein mutations for SARS-CoV-2 in the conservative regions. The violet box reflects RBD domain mutations. Most of the RBD mutations are located distally from RBM (except at position 417). The numbers are in accordance with the SARS-CoV-2 numbering scheme [17].

Figure 3 shows the aligned amino-acid sequences of S protein for MERS-CoV, SARS-CoV and SARS-CoV-2, and the mutation rates between SARS-CoV and SARS-CoV-2 for different amino-acid groups. Ten SARS-CoV-2 sequences from the US National Center for Biotechnology Information (NCBI) database (GenBank) were aligned by use of a genome alignment tool (Clustalw) and no differences in amino-acid sequences within the M and N proteins were found. However, there are two amino-acid variances in the S protein region. Additionally, two possible “L” and “S” SNPs have been detected in the ORF1ab and ORF8 regions. On the other hand, it has been shown that the addition of two stabilizing proline mutations in the C-terminal S2 fusion machinery has proved effective to stabilize some beta-coronavirus S proteins [15,28]. Studies regarding the biological symptoms of SARS-CoV-2 in experimental animals and humans will improve our understanding of the origin of the pandemic crisis [41]. The alignment of the RBM region of SARS-CoV-2 with those of the SARS-CoV and MERS-CoV viruses is shown in Figure 3A. Researchers argue that this similarity strongly suggests the convergent evolution of the SARS-CoV-2 and SARS-CoV RBD structures to improve binding affinity to the same ACE2 receptor, even though SARS-CoV-2 does not cluster within SARS-CoV in the beta coronavirus genus [6,42].

Several mutations have been reported in the conservative regions of the S protein of SARS-CoV-2 that differ from those in other SARS coronaviruses and SARS-like coronaviruses [17] (Figure 3C). Conserved regions are defined as regions that remain unchanged in other SARS and SARS-like coronaviruses. The SARS-CoV-2 RBD contains 195 residues that are traceable by density measurements. They are likely to be spread between ~T333 and P527, with N-linked glycosylation at asparagine (Asn343) (Figure 3A).

**Figure 4:**
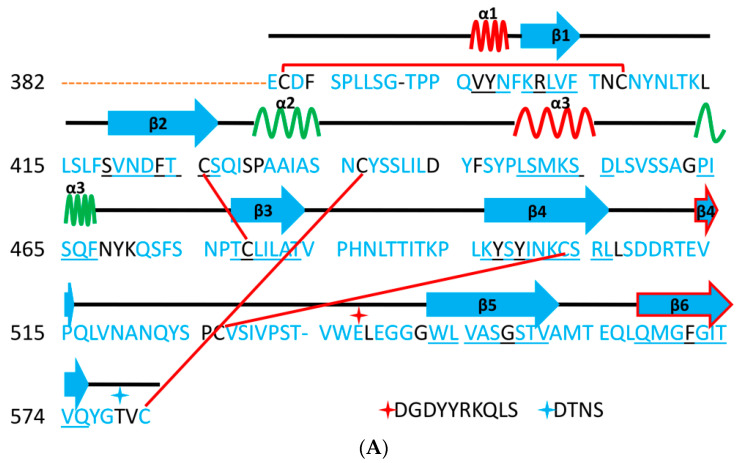

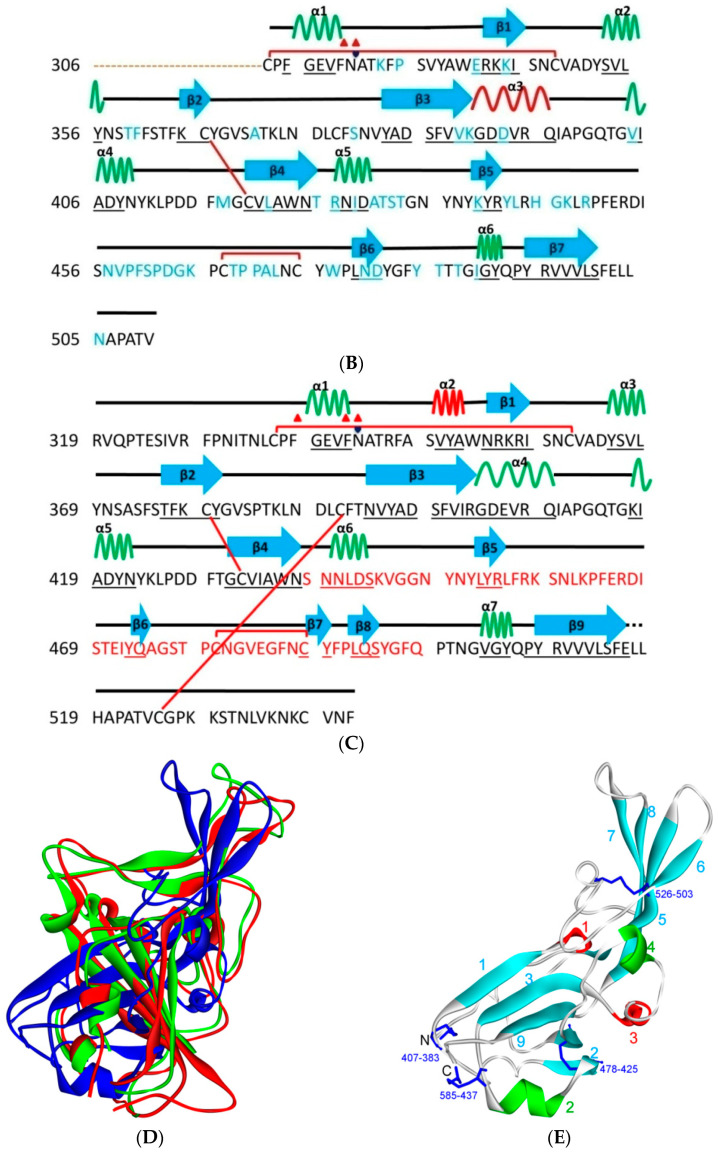

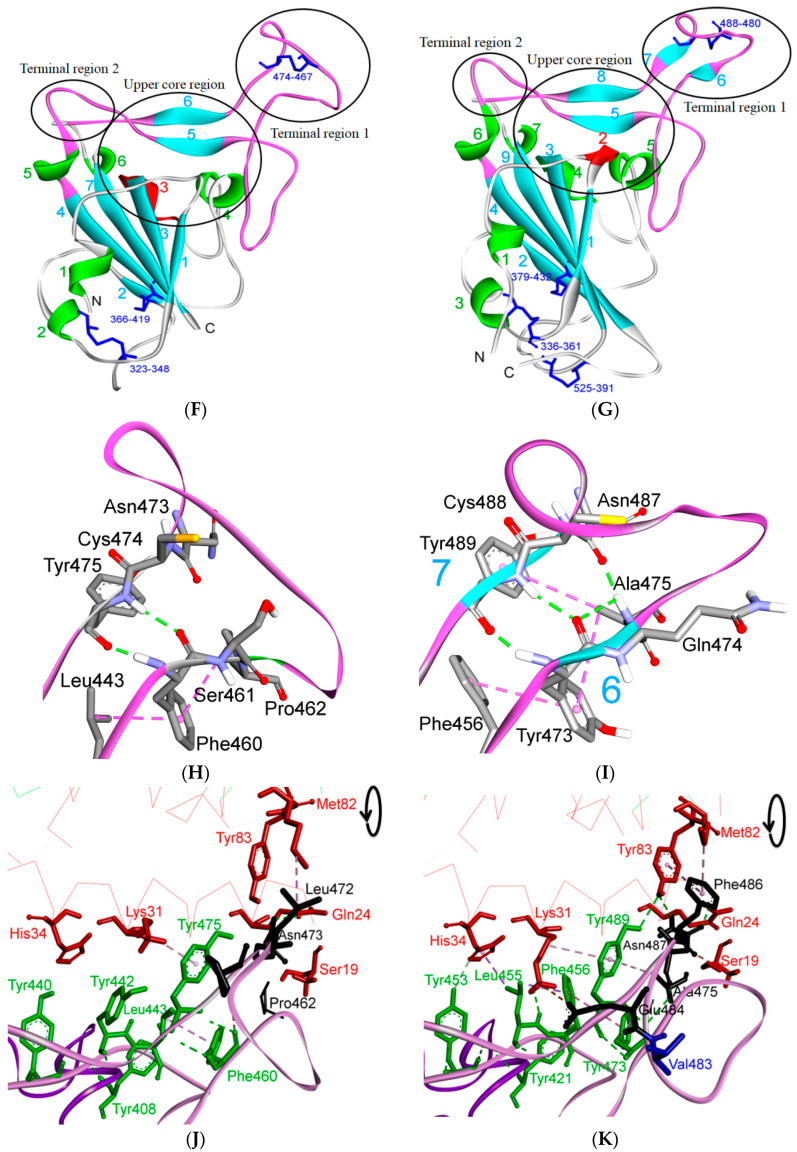
The aligned secondary structures of MERS-CoV, SARS-CoV and SARS-CoV-2, their distribution in the 3D structure and their influence on the binding contacts, and interactions with TR1. (**A**–**C**) are secondary structures of MERS-CoV, SARS-CoV and SARS-CoV-2, respectively. Disulphide bonds are represented by lines, and the sequences after the red and cyan stars in Figure 4A have been removed to keep the alignment formats with SARS-CoV-2. (**D**) The X-ray models of MERS-CoV (blue, PDB ID: 4L72), SARS-CoV (green, PDB ID: 2AJF) and SARS-CoV-2 (red, PDB ID: 6LZG) are structurally aligned. (**E**–**G**) show the 3D structures of MERS-CoV, SARS-CoV and SARS-CoV-2, respectively. (**H**,**I**) show parts of the RBM for both SARS-CoV and SARS-CoV-2, respectively. The influence of the differences in TR1 on the intermolecular contacts and on binding contacts with ACE2 is shown in (**J**) for SARS-CoV and (**K**) for SARS-CoV-2. In these diagrams, amino acids shown in green represent a hydrophobic pocket (preceding TR1) that shows slight differences between SARS-CoV and SARS-CoV-2. These differences may affect binding in this region (i.e., binding with Lys31 from ACE2) [6]. In all figures, β sheets, α-helices, and 3/10-helices are shown in cyan, green and red, respectively. Numbers in (**E**–**G**,**I**) indicate the order of secondary structures (i.e., β-sheets, α-helices, and 3/10-helices) in the S protein 3D model starting from the N-terminus. These numbers got the same color of the corresponding secondary structures. Underlined letters were used for those sequences with either β sheet or alpha helix conformation, while heart and triangle shapes indicate glycosylation and potential binding sites with different residues, respectively. These were based on the sequence analysis (April 2020) using protein data bank website (https://www.rcsb.org/). The blue sequences in (**A**,**B**) are those amino acids different from SARS-CoV-2. Red sequence in Figure 4C represents RBM region. 3D models were created using Discovery Studio (version 2.5.5, Biovia, San Diego, CA, USA).

Regarding the secondary structure of the S protein (Figure 4A–C), the SARS-CoV-2 RBD has a twisted, multi-stranded, anti-parallel β sheet (β1, β2, β3, β4 and β9) in which short connecting helices and loops act as the core. Between the β4 and β9 strands in the core, there is an extended insertion that contains short β5, β6, β7, and β6 strands, besides α5 and α6 helices and loops. This extended insertion is the RBM, which contains the majority of the SARS-CoV-2 contact residues that bind to ACE2. The RBD contains a total of nine cysteine residues, eight of which are resolved in the final model (based on X-ray crystallography, PDB ID: 6M0J, 2.45 Å) with four pairs of disulphide bonds. Of these bonds, three are in the core (Cys336-Cys362, Cys379-Cys432 and Cys391-Cys525), and these help to stabilize the β-sheets structure. The remaining disulphide bond (Cys480-Cys488) is found in the connecting loops in the distal region (terminal region 1 (TR1)) of the RBM. The locations of these disulphide bonds are similar in SARS-CoV and SARS-CoV-2, but they are slightly different from those in MERS-CoV [6]. The similar location of disulfide bond refer to a similar 3D structure, implies close functions and mechanisms of actions, of S protein for both SARS-CoV and SARS-CoV-2 (Figure 4F,G).

However, the SARS-CoV-2 RBD forms more atomic interactions with hACE2 than the SARS-CoVRBD does. This finding correlates with data that show higher affinity of the SARS-CoV-2 RBD (four folds) for receptor binding than is shown by the SARS-CoV RBD. The SARS-CoV-2 RBD binds to ACE2 with an affinity in the low nanomolar (nM) range; the dissociation constant (Kd) between ACE2 and the SARS-CoV-2 RBD is 4.7 nM, and between ACE2 and the SARS-CoV RBD is 31 nM, indicating that the RBD is the key functional component within the S1 subunit responsible for binding of SARS-CoV-2 by ACE2 [6,15,39,44].

### 5.1. Terminal Region 1

Based on the Protein Data Bank (PDB) website sequence analysis, the RBM of SARS-CoV-2 has a β sheet (β6 and β7) that is not found in SARS-CoV. Moreover, SARS-CoV-2 has an alanine residue (Ala475) that is located after β6, and which is involved in hydrophobic stabilizing interactions with β7 (Tyr489), and in a hydrogen bond with ACE2 (Ser19) [6]. At the same position in SARS-CoV, there is a proline residue, which leads to formation of a loop that causes a sharp kink in the loop structure. These differences affect the contacts between the RBMs of both SARS-CoV and SARS-CoV-2; SARS-CoV-2 makes stronger contacts with ACE2 in this area than SARS-CoV does [6,17,45] (Figure 4J,K).

The Phi (Φ; C-N-CA-C)/Psi (Ψ; N-CA-C-N) occurrences have been measured for the SARS-CoV RBD (PDB ID: 2AJF) and SARS-CoV-2 RBD (PDB ID: 6LZG). The dihedral angles are more confined to the preferred regions in the RBD of SARS-CoV-2 (for the SARS-CoV RBD domain, 84.1% of all residues are in favored regions vs. 94.3% of the corresponding residues in SARS-CoV-2), which may indicate a more stable RBD structure in SARS-CoV-2 compared with that in SARS-CoV [34,46,47]. Interestingly, the mean B isotropic value of the RBD of SARS-CoV-2 that is bound to ACE2 (PDB ID: 6LZG) is 44.01, which is lower than that (90.77) of the RBD of SARS-CoV (PDB ID: 2AJF). The B-factor can be seen as an indicator of the relative vibrational motion of various structural components. Low B-factor atoms belong to a well-ordered structure [43]. However, B-factors are greatly influenced by the resolution, and the variation in the resolution of the two X-ray structures may be the major contributor to this difference in the B-factor value [48].

Another interesting difference that has been observed in this region of SARS-CoV-2 compared with SARS-CoV is the insertion of Val483, which might increase the orderliness of this region [49]. This insertion could affect the druggability and vaccinability of the S protein. For example, it was found that in long molecular dynamics simulations (50 ns), lamivudine makes a favorable interaction profile with TR1; interactions (i.e., hydrogen bonding) were determined to be with Asn481, Val483 and Phe486 (Figure 4I,K). Moreover, it has been pointed in another study that residues of TR1 (called region IV in the study) include 9 residues (Cys480, Asn481, Gly482, Val483, Glu484, Gly485, Phe486, Asn487 and Cys488) that bind to GRP78 (a chaperone heat shock protein in cells) of the host [50]. While Cys480, Val483, Phe486 and Cys488 are described as active residues, critical for binding [51]. This region could also be targeted to inhibit the invasion of SARS-CoV-2 into the host cells via alternative pathways [50] (Figure 4I,K).

The hydrophobicity score of the IYQAG sequence (the initial part of the TR1) in SARS-CoV-2 is 5.6, while it is 8.7 for the corresponding part (PFSPD) in SARS-CoV. It is likely that the greater hydrophilicity of the IYQAG sequence in SARS-CoV-2 compared with its counterpart in SARS-CoV (Phe460 and Ser461) enhances the solvation with water (see the side chains of Tyr473 and Qln474 in SARS-CoV-2 (Figure 4H,I).

Regional differences in hydration dynamics around the protein surface have been connected with molecular recognition events within the protein. The tie between local solvent dynamics and regional protein dynamics (flexibility/stability) may explain protein conformational states, which are dependent on a measure of the local hydration and friction/viscosity parameters [52].

TR1 contains the PXXPP motif in SARS-CoV (PCTPP) (Figure 4B), while the corresponding part in SARS-CoV-2 is composed of PCNGV. Proline is an amino acid that is associated with rigid turns (hairpins). With the recent availability of an abundance of proteomic information, the importance of proline-rich regions has been demonstrated. Proline-rich regions are among the most common motifs that have been identified. Proline is markedly different to other amino acids, because it has an aliphatic side chain that is linked to nitrogen and α-carbon atoms. This cyclic side chain imposes conformational limitations that significantly affect the structures of proteins [53,54].

Moreover, PXXPP has been reported to be associated with disordered (unfolded) configurations (S score = 0.91; a value of S = 0.5 indicates a pattern with no preference for order or disorder, and values greater than 0.5 indicate a propensity towards unstructured configurations). Rather than adopting a single, well-defined structure, disordered proteins exist as an ensemble of native configurations. They often become folded upon binding to a target molecule. The intrinsic lack of structure seems to confer an advantage in these cases, and indeed, disorder appears to be conserved through evolution [55,56].

### 5.2. Terminal Region 2

**Figure 5:**
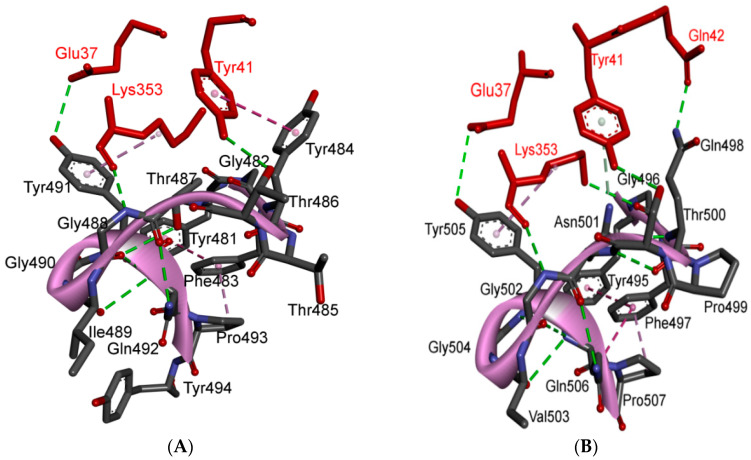

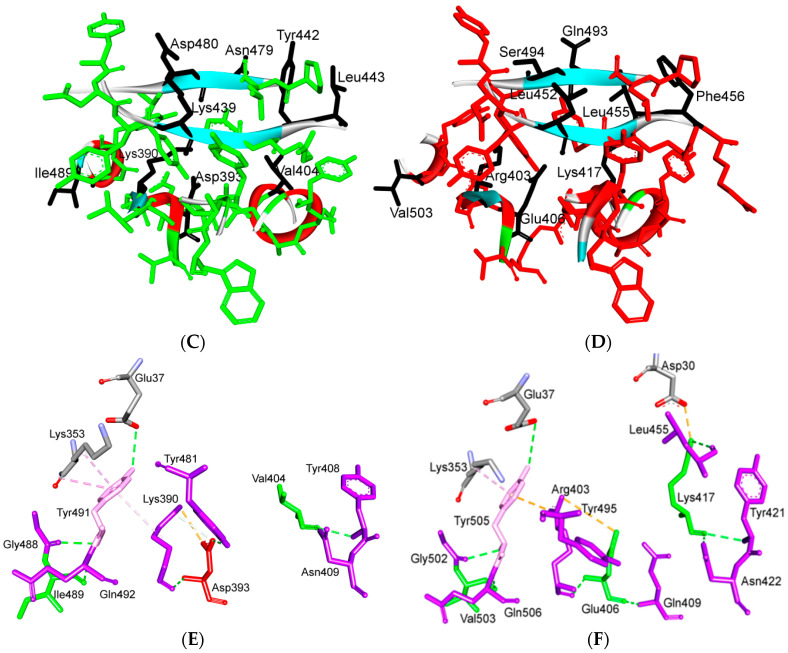
Structure of TR2 and ridge regions in both SARS-CoV and SARS-CoV-2, and their interactions to ACE2. (**A**,**B**) show the intra-molecular and inter-molecular contacts of TR2 region in SARS-CoV and SARS-CoV-2, respectively. The number of the interactions in TR2 region is noticeably higher in SARS-CoV-2 compared to that of SARS-CoV. The presence of Asn501 in SARS-CoV-2 (instead of Thr487) makes a substantial difference since it is involved in stacking with Tyr41 on ACE2. This stacking liberates Gln498 (instead of Tyr484 in SARS-CoV) to make a strong hydrogen bond with Gln42 of ACE2. (**C**,**D**) show the environment of the ridge and upper core regions of SARS-CoV and SARS-CoV-2, respectively. The residues that are labelled in black show the amino acids in this region that differ between SARS-CoV and SARS-CoV-2. (**E**,**F**) show the main amino acids in the upper core region. The green and red residues indicate those in α-helices and 3/10 helices in this region, respectively. These amino acids are in contact with other amino acids in the RBM (pink) and RBD (violet). Lys417 is the main player in this region, since it is involved in electrostatic interactions with Asp30 from ACE2. 3D models were created using Discovery Studio (version 2.5.5, Biovia, San Diego, CA, USA).

At the other end of the S protein, terminal region 2 (TR2) also undergoes critical interactions with ACE2 [6]. This hotspot is characterized by its rich glycine content (GFYTTTGIG in SARS-CoV vs. GFQPTNGVG in SARS-CoV-2). The presence of proline in the middle of TR2 in SARS-CoV-2 may affect the behavior of this region. Two motifs have been determined in this region (GXXP and PXXG) of SARS-CoV-2, both of which have been associated with sharp kinks [57] that may force the surrounding regions to move outward or inward and thus to affect protein-protein interactions. For example, one hallmark of G-protein coupled receptor (GPCR) activation is the outward movement of TM6 that is accompanied in Class B GPCRs by the formation of a sharp kink in the middle of the transmembrane domain at the highly conserved PXXG motif (Figure S1 and Figure 5A,B).

The blocking of two positions (Gln498 and Asn501) in this region has been found to offer critical advantage in the design of antiviral peptides that are superior to conventional drugs and may also be effective against SARS-CoV-2. It has been found that the DBP6 peptide, which has been identified from databases, can block the interaction of sites on the SARS-CoV-2 RBD with different amino acids; one critical position is that of Gln498. Gln498, Thr500 and Asn501 of the RBD form a network of hydrogen bonds with Tyr41, Gln42, Lys353 and Arg357 from ACE2 [45]. Moreover, it was reported that Arg426 in this region for SARS-CoV forms a strong electrostatic interaction with Glu329 of ACE2, while this interaction was not seen in SARS-CoV-2 (where arginine is replaced with asparagine), which makes this region more favorable for SARS-CoV compared to SARS-CoV-2. Other changes (Tyr484 and Thr487 in SARS-CoV to Gln498 and Asn501 in SARS-CoV-2, respectively) may have lower effect on the difference between binding affinities in TR2 between the two viruses, since they involve in forming hydrogen or stacking interactions of nearly equivalent energies [2,40,45] (Figure S1 and Figure 5A,B).

### 5.3. Middle Ridge of RBM and Upper Core Region of RBD

This segment of the S protein reinforces the interaction of the SARS-CoV-2 RBD with amino acids through the engagement of two polar residues [45,56] (Figure 5D). In the core region of SARS-CoV-2, there is a unique residue (Lys417) that interacts with ACE2. It forms salt-bridge interactions with Asp30 of ACE2. This position is substituted by a valine amino acid in the SARS-CoV RBD. The valine amino acid interrupts the binding with ACE2 (Figure 5E,F). Furthermore, a comparison of the surface electrostatic potentials between the viruses has also identified a positively charged patch on the SARS-CoV-2 RBD, which is contributed by Lys417 and which is absent on the SARS-CoV RBD [6]. This residue is located in the middle of the ridge [45] and might contribute significantly to the stability of the core region of the RBD and thus affect the affinity to bind with ACE2. The Val-to-Lys417 mutation in the receptor-binding domain of SARS-CoV-2 is reported to hinder the neutralization of SARS-CoV-2 by anti-SARS-CoV mAbs [45] (Figure 5C–F).

It has been noted that positions Lys417 and Gly502 (identical conservative residues between SARS-CoV and SARS-CoV-2) provide two of the strongest binding impacts (binding is reduced by 78% and 79% upon mutation of these positions to Ala, respectively). This is because Lys417 and Gly502 help to establish a strong electrostatic contact with Asp30, and other contacts with Gln325, Lys353 and Gly354 [58]. Tyr505, which corresponds to Tyr491 in SARS-CoV, is acritical conserved amino acid as it forms strong interactions with surrounding amino acids (i.e., Pi-cation interaction with Arg403 in the SARS-CoV-2 RBD, Pi-alkyl interaction with Lys353 of ACE2 and hydrogen bonding with Glu37 of ACE2) (Figure 5E,F).

**Figure 6:**
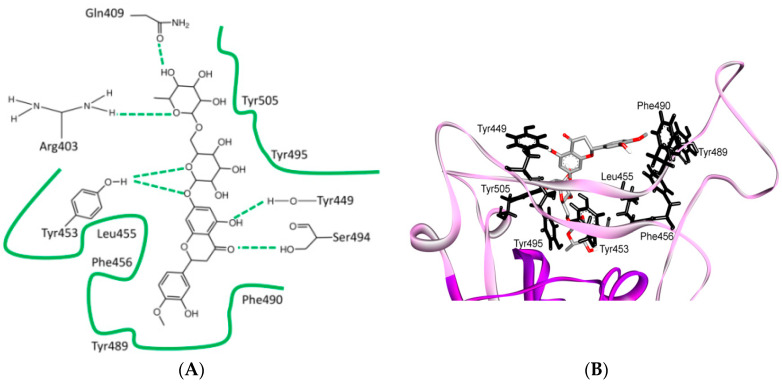

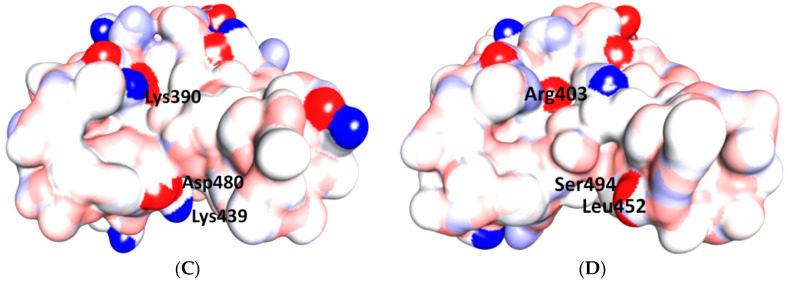
Differences of shallow pit in the ridge region of RBD for SARS-CoV and SARS-CoV-2, and its binding with hesperidin in SARS-CoV-2. (**A**) Two-dimensional structure of hesperidin and its surrounding interactions with the SARS-CoV-2 RBD. (**B**) Hesperidin has been predicted to lie on the middle shallow pit of the surface of the RBD of the S protein, where the dihydroflavone part of the compound lies parallel with the β6 sheet of the RBD. The sugar moiety is inserted into the shallow pit in the direction away from ACE2, where a few hydrophobic amino acids, including Tyr449, Try453, Leu455, Phe456, Phe490, Try489, Try495 and Tyr505 (corresponding to Tyr436, Tyr440, Tyr442, Leu443, Trp476, Tyr475, Tyr481 and Tyr491 in SARS-CoV) form a relatively hydrophobic shallow pocket to contain the compound. Hydrogen-bonding interactions have been predicted between Tyr453, Ser494, Gln409, Tyr449, Arg403 (corresponding to Tyr440, Asp480, Gln396, Tyr449 and Lys390 from SARS-CoV) and the compound. Tyr453 and Tyr449 are among the critical amino acids. (**C**,**D**) in the space-filling models of the S protein in SARS-CoV (left) and SARS-CoV-2 (right), the shallow pit is apparent (Tyr453 and Tyr449 are among the critical amino acids). The hydrophobic cleft is apparently similar in both viruses; the differences are mainly located at the gates of this cleft, where Asp480 and Lys439 at one entrance in SARS-CoV are replaced by Ser494 and Leu452 in SARS-CoV-2, while Lys390 at the other entrance in SARS-CoV is replaced by Arg403 in SARS-CoV-2. These changes may affect the binding affinities of proposed drugs with SARS-CoV and SARS-CoV-2. It has been reported that the electrostatic potential observed for SARS-CoV-2 is more positive than that for SARS-CoV, which is consequently considered to result in a greater electrostatic interaction between ACE2 and SARS-CoV-2. This could be explained by the replacement of the negatively charged amino acid Asp by a neutral Ser (Asp480 to Ser494) [59]. Three-dimensional models were created using Discovery Studio (version 2.5.5, Biovia, San Diego, CA, USA).

Other than Lys417, the other two mutations (Ile to Val503 and Asp to Glu408) are expected to have a lesser effect since the mutating groups have the same properties and interaction contacts as the residues they replace (Figure 5C–F). A shallow pit in the middle ridge is expected to offer a binding interface target for different drugs. It is believed that the S protein should be useful for further structure-based virtual screening and related computer-aided drug development and vaccine design [59]. On the basis of virtual screening results that have been published in one study, most investigated compounds are not predicted to bind with the binding interface of the spike-ACE2 complex. The only compound that could target the binding interface between spike and ACE2 is hesperidin, which lies on the central shallow pit on the surface of the middle ridge of the RBD (Figure 6).

### 5.4. Other Regions of S Protein

The most drastic difference between the S protein between viruses in other regions of the S1 domain is the presence of an additional N-glycosylation site at Asn370 on SARS-CoV-2 (Asn357 in SARS-CoV). The N-glycan sequence (N-X-S/T, in which X is any amino acid but proline) arises due to an amino-acid difference at residue 372, where SARS-CoV has a Thr, but SARS-CoV-2 displays an Ala [60]. Mass spectrometric analysis shows that a complex glycan is indeed present at this N-glycosylation site in SARS-CoV [60].

Aside from the well-defined RBM regions, a new type of ACE2 ganglioside-binding domain at the tip of the N-terminal domain of the SARS-CoV-2 S protein has been identified [61]. This domain (spanning the region from position 111 to position 158), is fully conserved among clinical isolates worldwide, is believed to improve attachment of the virus to lipid rafts and facilitate its probability of subsequent attachment to the ACE2 receptor [61]. Mutations in the S protein may partially explain the discrepancy between the predicted and actual number of deaths caused by SARS-CoV-2, and may explain that other factors aside from social distancing, such as competition between two strains of differing virulence, may be at play. A sequence analysis from 2310 viral isolates from Nextstrain reveals that the residue at position 614 of the viral spike protein is changed from a putative ancestral aspartic acid (D) to a glycine (G) between two viral clades. The G strain is predominantly found on the east coast of the United States, and the D strain is predominantly found on the west coast. Point mutations in a coronavirus spike protein can result in increased virulence through instability of the viral machinery and altered viral-to-cell-membrane fusion [62].

## 6. Is ACE2 the Receptor for S Protein?

It has been reported that the spike glycoprotein of SARS-CoV-2 is modified via homologous recombination and is a mixture of bat SARS-CoV and an unknown Beta-CoV [3,32]. Superimposition of the cryo-EM structure of the RBD of SARS-514 CoV-2 onto a previously reported SARS RBD structure revealed a root-mean-square deviation (RMSD) of 3.8 Å over 959 Cα atoms [2], which indicates highly similar 3D protein structures [63,64,65].

Sequence similarities between SARS-CoV-2 and SARS-CoV spikes imply that they share the same ACE2 receptor. Importantly, compared with the SARS-CoV RBM, the SARS-CoV-2 RBM does not contain any deletion or insertion, except for a single-residue (Val483) insertion in TR1 (Figure 3A and Figure 4K). This information provides additional evidence that SARS-CoV-2 uses ACE2 as its receptor [1,66]. Other evidence that ACE2 is the receptor for SARS-CoV-2 is that, among the 14 ACE2 contacting residues in the RBD, nine are fully conserved and four are partially conserved in SARS-CoV-2 and SARS-CoV (Figure 3) [1,27].

**Figure 7:**
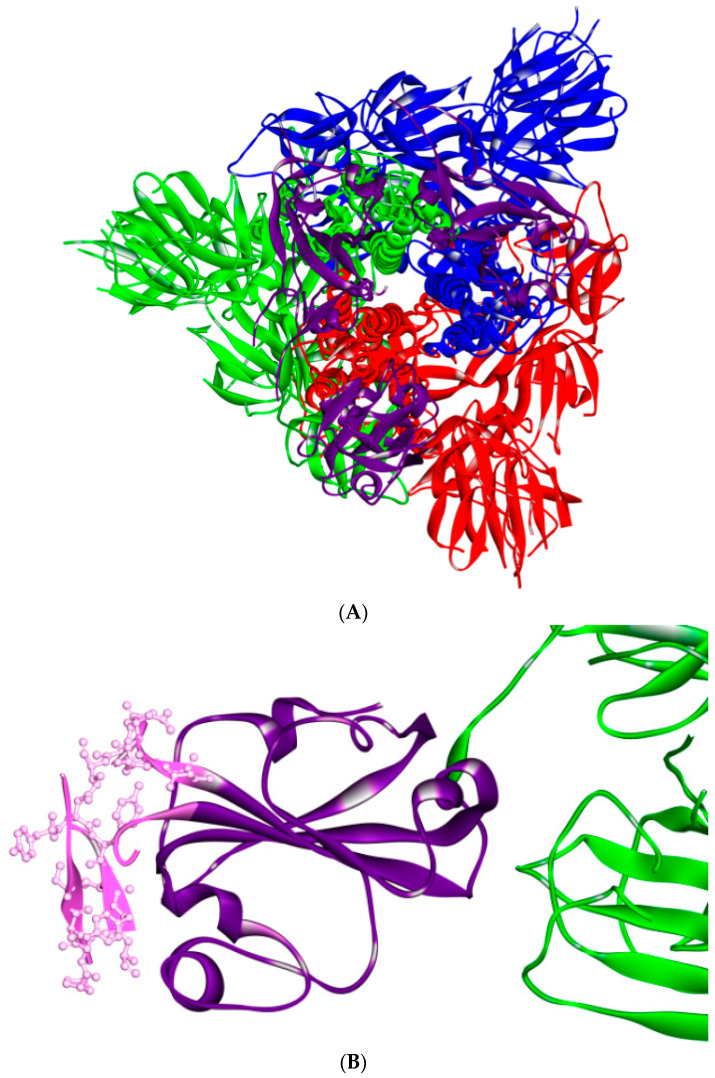
Structure of S protein (PDB ID: 6vsb) [14]. (**A**) RBD is shown in violet. (**B**) Close side view of RBD in violet, with receptor-binding motif (RBM) annotated in pink. Three-dimensional models were created using Discovery Studio (version 2.5.5, Biovia, San Diego, CA, USA).

However, it has been reported that there are no data on the relationship between ACE2 activity and SARS-CoV-2 mortality [67]. On the other hand, it has been reported that some cell types in the host that express ACE2 beyond lung epithelial cells, are not attacked by the SARS-CoV-2 virus, while cells that lack ACE2 can bind the SARS-CoV-2 virus [68]. The differences in S protein may have implications for the virulence of SARS-CoV-2 and its binding affinity, which will affect vaccine and therapy development; for example, polyclonal anti-SARS S1 antibodies T62 inhibit entry of SARS-CoV S, but not SARS-CoV-2 S pseudo-virions [69]. The S protein is a homotrimeric glycoprotein fusion class I (Figure 7), which, in a metastable conformation prefusion, undergoes a substantial structural rearrangement in order to fuse the viral membrane and the host cell membrane [2,15,27,70,71,72,73].

This process is triggered when the S1 subunit binds to a host-cell receptor. Receptor binding destabilizes the pre-fusion trimer (in which receptor binding to RBDs leads to an unstable RBD up-conformation) and the result is the shedding of the S1 subunit and transition of the S2 subunit to a stable post-fusion conformation. It has been shown that the RBD of SARS-CoV-2 S1 undergoes hinge-like conformational movements (which are also a feature that is found in SARS-CoV, MERS-CoV and some alpha-coronaviruses) that transiently hide or expose the determinants of receptor binding. These two states are referred to as the “down” conformation and the “up” conformation, in which the “down” conformation corresponds to the receptor-inaccessible state [15]. The ACE2 host receptor can only interact with the RBD when it is in the up conformation, since the down conformation is inaccessible to ACE2 [60]. Moreover, SARS-CoV-2 has been shown to use the TMPRSS2 serine protease to prime the S protein. A TMPRSS2 inhibitor has been shown to block entry; it has been approved for clinical use and might constitute a treatment option [39,74].

There is no evidence that S protein immunogenicity or its link with the ACE2 receptor is affected by either the absence or the presence of other structural proteins. This condition is a critical initial stage for viruses to access the host cell [75]. Differences in the S protein affect recognition by the ACE2 receptor and other proteins or ligands. For example, it was found that a panel of monoclonal antibodies and murine polyclonal antisera against SARS-S1/RBD are unable to bind to the SARS-CoV-2 S protein [2]. This finding suggests that researchers must investigate the previously developed SARS-RBD-based vaccine candidates for any clinical benefit for SARS-CoV-2 prophylaxis.

The position of RBDs in their respective down conformations is one of the significant differences between SARS-CoV and SARS-CoV-2S1 structures (although a relatively small difference). The SARS-CoV RBD is closer to the NTD of the neighboring protomer in the down conformation than in the up, whereas the SARS-CoV-2 RBD is placed at a sharp angle into the down conformation towards the central cavity of the trimer. However, when the individual structural domains of SARS-CoV-2 are aligned with their counterparts from the SARS-CoV S, they reflect the high degree of structural homology between the two proteins [15].

## 7. Differences in Furin-Like Protease Recognition Pattern

Seven pro-protein convertases (PCs) cleave precursor proteins at specific single or paired basic amino acids within the motif (R/K)-(2X)n-(R/K)↓(canonically, R-X-(R/K)-R), in which n = 0, 1, 2 or 3 spacer amino acids [1,76]. PCs, in particular furin [77,78,79], have been involved in viral infections due to their role in the processing of many critical surface-cell proteins.

**Figure 8:**
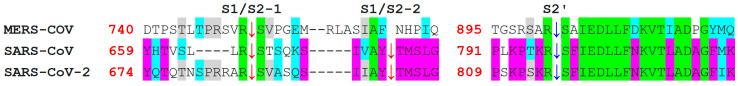
Aligned sequences (performed by use of Clustalw) of furin-like cleavage sites for SARS-CoV-2 that are aligned with those of SARS-CoV and MERS-CoV. Green color indicates positions that have a single, fully conserved residue between the three sequences. Pink indicates amino acids that are conserved between SARS-CoV-2 and SARS-CoV, while grey color indicates those that are conserved between SARS-CoV-2 and MERS-CoV. Cyan indicates conservation between groups that exhibit strongly similar properties and score > 0.5 in the Gonnet PAM 250 matrix [85]. The red arrowheads indicate S1/S2 possible cleavage sites, while the blue arrow shows a S2′ cleavage site. The first S1/S2 site is very similar between SARS-CoV-2 and MERS-CoV, while the second S1/S2 is found only in SARS-CoV and SARS-CoV-2. The S2′ cleavage site in SARS-CoV-2 is similar to that of SARS-CoV.

**Figure 9:**
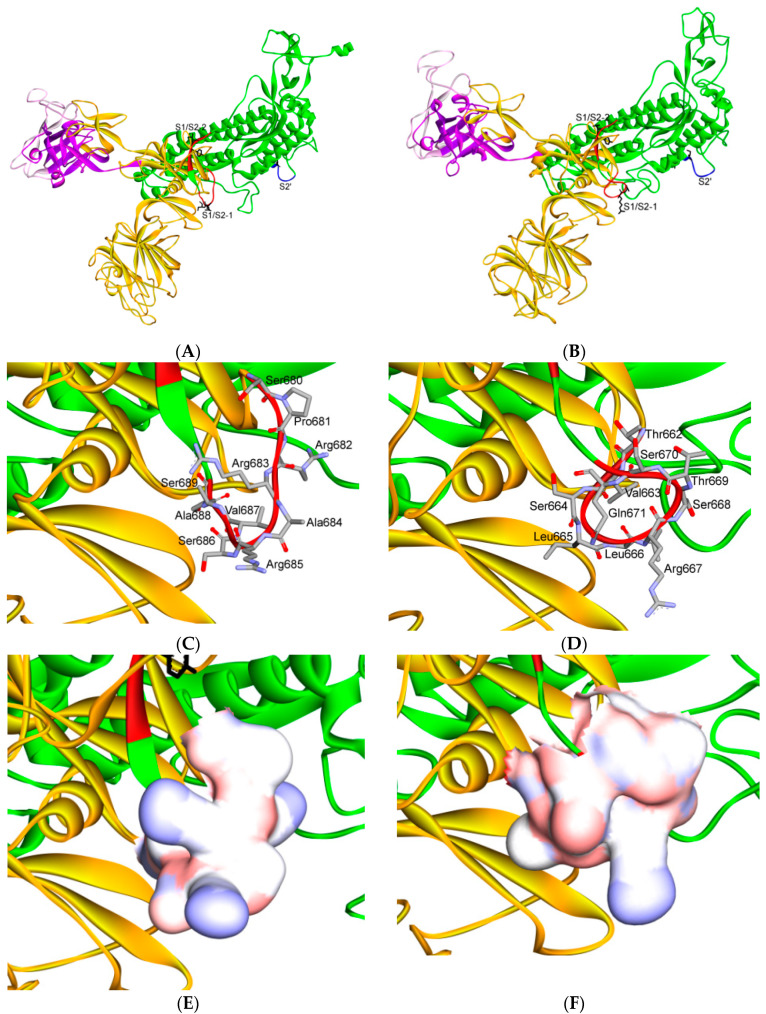
Three-dimensional structure of different furin-like cleavage sites for SARS-CoV and SARS-CoV-2. (**A**,**B**) show 3D models of the S protein of SARS-CoV and SARS-CoV-2, respectively. These have been predicted by homology modeling that used Swiss-model software, based on the S protein sequence obtained from Genbank (accession numbers: MT192773 and AY278741 for SARS-CoV and SARS-CoV-2, respectively). The models were predicted through use of PDB IDs 6ACD and 6VYB for SARS-CoV and SARS-CoV-2, respectively. The S2 unit, RBD, RBM, S1/S2 and S2′ potential cleavage sites are shown in green, violet, pink, red and blue, respectively. The S1/S2-1 is more exposed to solvent than the S1/S2-2 site, so it is more easily accessed and cleaved by furin in SARS-CoV-2. Therefore, it is a more potential effective priming site. (**C**,**D**) show the detailed amino acid compositions of the S1/S2-1 cleavage sites of SARS-CoV and SARS-CoV-2, respectively. (**E**,**F**) show the surface of the S1/S2-1 cleavage sites of SARS-CoV and SARS-CoV-2, respectively. There are more basic groups in the S1/S2-1 cleavage site of SARS-CoV-2 (shown in light blue color) than in that of SARS-CoV. 3D models were created using Discovery Studio (version 2.5.5, Biovia, San Diego, CA, USA).

Furin is used by a number of pathogens in other roles. For example, the envelope proteins of viruses such as human immunodeficiency virus (HIV), influenza, dengue fever, several filoviruses, including ebola and marburg viruses, and possibly the spike protein of SARS-CoV-2 [1,77,78,79], must be cleaved by furin or furin-like proteases to become fully functional. Inhibitors of furin are under consideration as therapeutic agents to treat anthrax infection [80]. ProP [81] and PiTou [82] are two bioinformatics methods that can be used to predict furin substrates and locations of furin cleavage sites in protein sequences. The S1/S2 processing sites exhibit different motifs among coronaviruses; many of them display cleavage sites after a basic residue. It is, thus, likely that the priming process is ensured by different host-cell proteases, the choice of which depends on the sequence of the S1/S2 cleavage site. Accordingly, the MERS-CoV S protein that contains the RSVR↓SV motif can be cleaved by furin during viral egress [83,84]. The SARS-CoV-2 S protein contains a putative furin recognition motif (PRRARSV) (Figure 8 and Figure 9) that is similar to that of MERS-CoV.

It has been shown that the spike protein is cleaved at the S1/S2 cleavage site to generate the subunits S1 and S2. Many viruses display the canonical (R/K)-(2X)n-(R/K)↓ motif (Figure 8). Further, the variation around the viral envelope glycoprotein cleavage site has been shown to play a role in cellular tropism and pathogenesis [76,77,78,79]. Conversely, the S protein of SARS-CoV remains largely uncleaved after biosynthesis, possibly due to the lack of a favorable furin-like cleavage site (SLLRST). In this case, it has been reported that, after receptor binding, the virus coerces the proteases of target cells such as elastase, cathepsin L or TMPRSS2 to cleave the S protein at a conserved sequence AYT↓M (located 10 amino acids downstream of SLLRST). As the priming event is essential for virus entry, the efficacy and extent of this activation step by the proteases of the target cells should regulate cellular tropism and viral pathogenesis. In the case of the SARS-CoV-2 S protein, the conserved site 2 (S1/S2-2) sequence AYT↓M may still be cleaved, possibly after the preferred furin cleavage at the site 1 [27]. The 3D model of the three cleavage sites of SARS-CoV and SARS-CoV-2 are shown in Figure 9.

Strikingly, the SARS-CoV-2 S protein sequence contains 12 additional nucleotides upstream of the single Arg↓ cleavage site 1 (S1/S2-1) compared with the same site on the SARS-CoV S protein. This addition forms a predicted solvent-exposed PRRAR↓SV sequence, which corresponds with a canonical furin-like cleavage site (Figure 8 and Figure 9) [27,86,87,88]. This furin-like cleavage site should be cleaved during virus egress [83] in order to promote S protein “priming” and it may provide a gain-of-function to the SARS-CoV-2 virus for efficient spread in the human population, compared with other lineage b beta coronaviruses. If this site is not processed, it is expected that the S protein is cleaved at site 2 during virus endocytosis, as has been observed for the SARS-CoV virus. The furin-like S2′ cleavage site at KR↓SF, which contains P1 and P2 basic residues and a “P4” hydrophobic Phe [83], is identical between SARS-CoV and SARS-CoV-2.

In the MERS-CoV S protein, the sequence is replaced by RXXR↓SA at the S2′ site, where P1 and P4 are basic residues and an alanine is found at P6. This sequence suggests a less favorable cleavage by furin. However, processing at S1/S2-1 in SARS-CoV-2 is expected to be a key part of the final activation of the S protein. These observations suggest that inhibitors of furin-like enzymes may contribute to the inhibition of virus propagation [27].

A comparable type of modification (insertion) alters the H5N1 influenza virus and other avian viruses into more pathogenic variants. The origin of the high pathogenicity of avian influenza H5N1 due to the RRRKK insertion at the cleavage loop of the haemagglutin in H5 has been studied and compared with those of the non-inserted H5 and H3 that are bound to the furin active site. The highly pathogenic H5 cleavage loop has been found to be strongly bound to the furin cavity as a proteolytic conformation. Experimentally, the RRRKK insertion also increases the amount of haemagglutinin cleavage by furin [89].

Moreover, the RRXR sequence has been shown by many studies to be a putative furin cleavage site. TGF-β has been shown to be cleaved from its pro-peptide by furin-like endo-proteinases at the RRXR sequence during secretion [90]. Additionally, in the ADAM metallo-peptidase domain 17 (ADAM17), also called TACE, the pro-domain is cleaved from the catalytic domain at the RRXR site [91]. MT1-MMP is synthesized as a latent zymogen. The zymogen requires proteolytic cleavage of the N-terminal prodomain peptide sequence [92,93]. The main activation mechanism of MT1-MMP has been suggested for cleavage at one or both RRXR-furin sites [94].

Dahms et al. have searched for inhibitory peptides that have similar sequences to that of the S1/S1-1 cleavage site [76]. They have reported the inhibition activity of three different peptides, namely: I1: H-Arg-Arg-Arg-Val-Arg-4-aminomethyl-benzamidine (R-R-R-V-R-X1); I2: phenylacetyl-citrulline-Arg-Val-Arg-4-aminomethyl-benzamidine (X2-R-V-R-X1); and I3: 4-aminomethyl-phenylacetyl-Arg-Tle-Arg-4-aminomethyl-benzamidine (X3-R-tL-R-X1). Their inhibition constants were reported to be 33.7 pM, 238 nM and 22.4 pM, respectively; I1 and I3 were the most active [76].

Inhibitory peptide I1 has a core sequence (RRXR) that is closely related to that of the S1/S2-1 cleavage site of SARS-CoV-2 (R-R-A-R), with the X position filled by a hydrophobic, small amino acid. Interactions of I1 that have been observed at the Arg3 position involve typical hydrogen bonds with Tyr308 and charge-assisted hydrogen bonds with Asp264 and Glu236. The guanidino group of the Arg2 residue also mediates electrostatic interactions with Glu236. Additional Arg2 interactions include hydrogen bonds to the carbonyloxygen of Val231 and to a specific water molecule that is tightly bound by the side chains of Asp233 and Glu236 and by the amide nitrogen of Ala267 [76].

**Figure 10:**
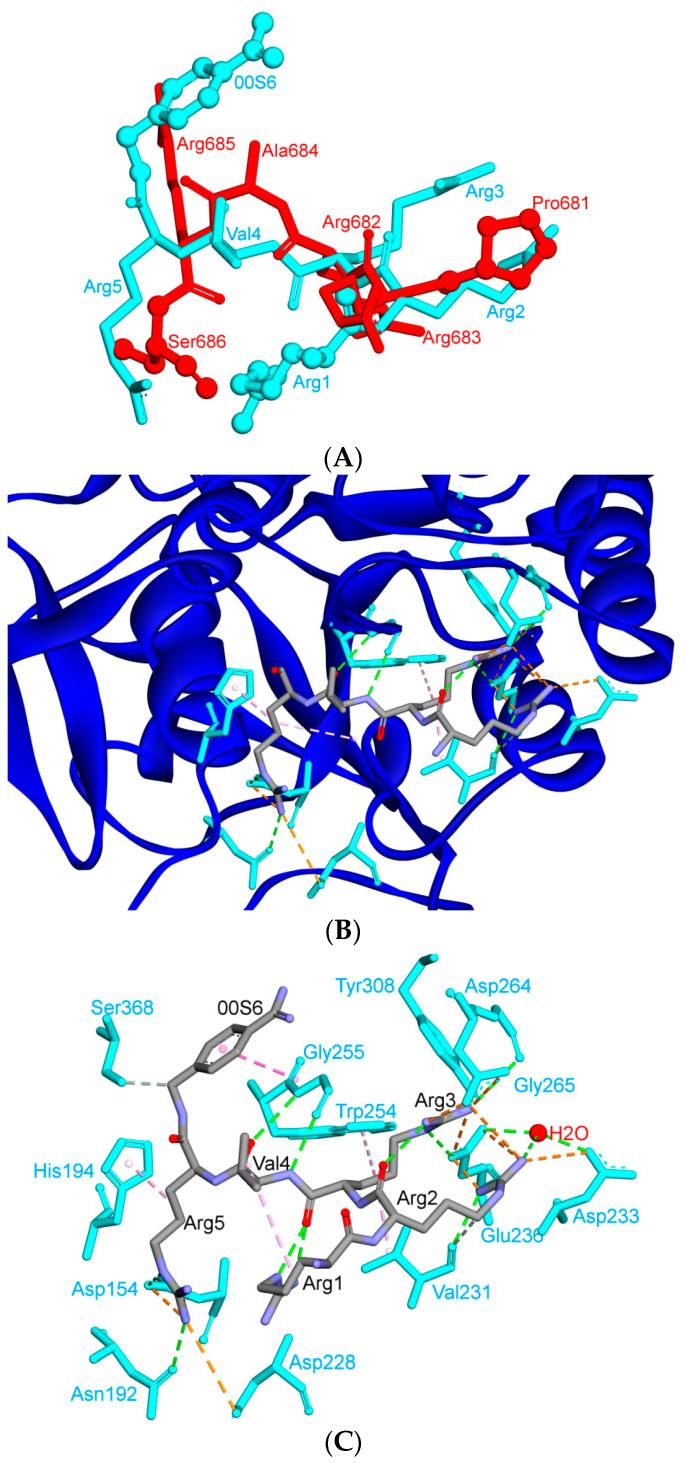
S1/S2-1 furin cleavage site and its similarity to the I1 inhibitory peptide. (**A**) The alignment of the SARS-CoV-2 S1/S2-1 site in SARS-CoV-2 with the I1 peptide. (**B**) The binding mode of the furin active site with the I1 inhibitory peptide. (**C**) The interactions of the I1 inhibitory peptide with the amino acids of the furin active site; same interactions are expected with the S1/S2-1 cleavage site. Three-dimensional models were created using Discovery Studio (version 2.5.5, Biovia, San Diego, CA, USA).

An intra-molecular hydrogen bond has been observed between the Arg2 carbonyloxygen and the Arg3 guanidino group. As a result of the extensive interaction network, the backbone conformation of this peptide bond is largely restricted. This backbone conformation of the inhibitor is also stabilized by water-bridged interactions between the Arg2 carbonyloxygen and the amide nitrogen of Glu257 as well as between the Arg2 amide nitrogen and the amide nitrogen of Val231 (Figure 10) [76].

The RRXR sequence (POS-POS-X-POS) in general has an S score of 0.84 [55], which means it is disordered, but in the structure of S1/S2-1, the backbone might be affected by the presence of proline (Pro681) upstream of the cleavage site, which is likely to increase the amount of order in the site.

The bulky Arg1 residue of I1 (which corresponds to Pro681 in the S1/S2-1 site) replaces water molecules from the active site cleft of furin (which are believed to be replaced by flanking amino acids in the S1/S2-1 cleavage site of SARS-CoV-2). Several ordered solvent molecules have been observed to bind in close proximity to Arg1 in structures that involve inhibitors that lackan Arg1 residue. Some of these water molecules show conserved binding sites. The liberation of bound solvent molecules by the inhibitor at Arg1 is, hence, expected to increase the entropy of binding and therefore productively contribute to the binding energy.

More interestingly, the PRRXR sequence (which is equivalent to the S1/S2-1 cleavage site of SARS-CoV-2) has been reported to be apat7 nuclear localization signal (NLS). The pat7 NLS starts with proline and is followed within three residues by a segment that contains three basic residues out of four. The presence of this NLS has been confirmed by use of the computer program PSORT [95]. Even though replication of many viruses is restricted to the cytoplasm, the N proteins of several viruses have been reported to localize to the nucleolus during infection by NLSs [96,97,98,99]. Viral proteins have the capacity to modulate the nucleolar function, and this modulation can be a viral strategy that diverts biosynthetic resources from the dividing nucleus to the cytoplasm, the site of virus replication [44,85,99,100,101]. The ability of S protein in the cytoplasm to perform such a function requires verification [102].

## 8. Effect of the S Protein and ACE2 Differences on the Binding Contacts, Affinity and Virulence

It has been shown that in SARS-CoV-2, the presence of glutamine, asparagine, leucine, phenylalanine and serine amino acids enhances ACE2 binding [103]. Several studies have proposed that the increased virulence of SARS-CoV-2 compared with that of other coronaviruses is due to its higher binding affinity to the ACE2 receptor [1,45,85,104]. Yan et al. have proposed that the mutation of Val404 to Lys417 may result in a higher binding affinity, due to the salt bridge between K417 and D30, whereas the mutation of Arg426 to Asn439 would weaken the interaction with Glu329 [45]. However, it was shown by simulations that the Val404 to Lys417 mutation increased the binding affinities, due to favorable electrostatic interactions that were greater than the energy losses that were induced by the Arg426 to Asn439 mutation.

Additionally, it was proposed that the mutation of Leu472 to Phe486 would weaken the Van der Waals (vdw) contact with Met82 of ACE2 [45]. It was found that two main residues (Asn479 and Asn487) were associated with hACE2 [105], and enabled virus progression and tropism [11]. In addition, a single mutation in Asn501 position has been found to enhance the binding capacity of the SARS-CoV-2 RBD with the human ACE2 and this evolution in infected patients should be monitored [38,44,105].

Nearly 20-fold increased binding energy between ACE2 and the SARS-CoV-2 spike trimer has been reported (KD of 14.7 nM), compared with that between ACE2 and the SD1 site of the SARS-CoV RBD (KD of 325 nM). This is perhaps due to experimental issues, such as the different proteins that were used in the assay. Nevertheless, the binding affinity alone is unlikely to explain the unusual transmissibility of SARS-CoV-2. Other factors such as the unique RRAR furin cleavage site at the S1/S2 boundary of the SARS-CoV-2 spike may play more important roles in the facilitation of rapid human-to-human transmission [6,39,44,82,85].

**Figure 11:**
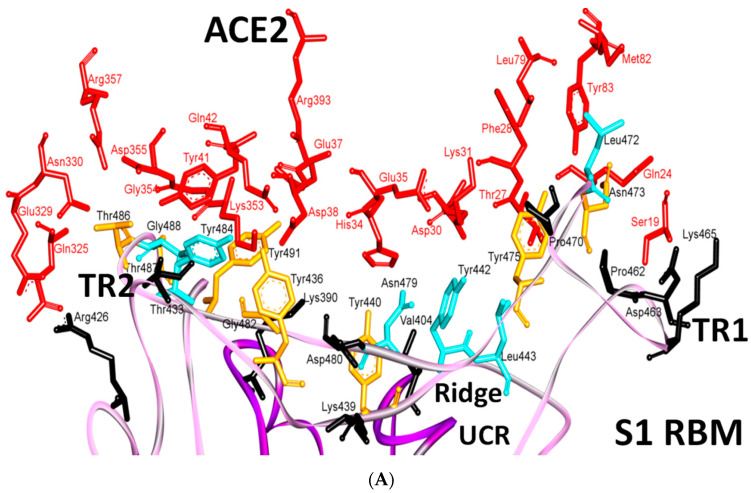

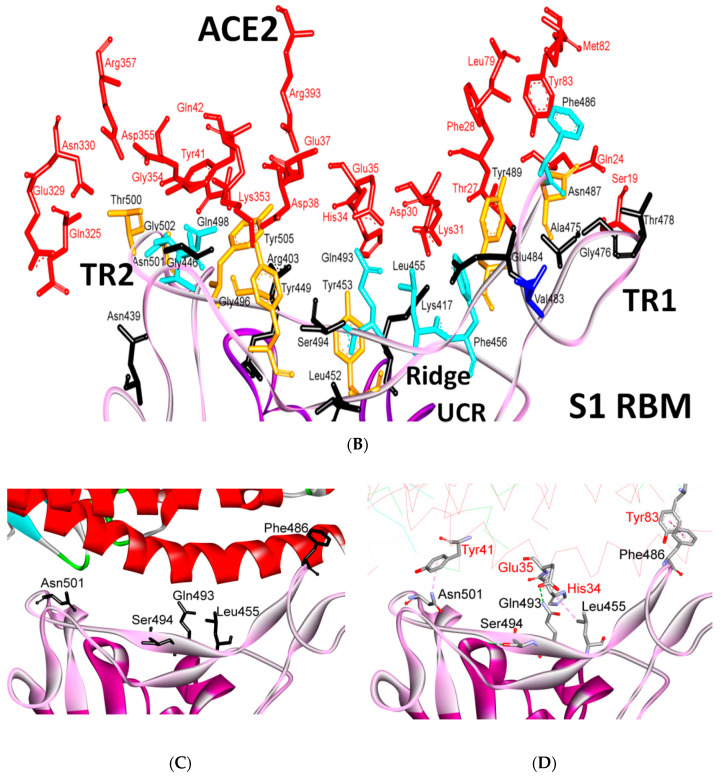
Binding interface and interactions between RBM of SARS-CoV and SARS-CoV-2 with ACE2. (**A**,**B**) interacting residues between the RBM and ACE2 for SARS-CoV and SARS-CoV-2, respectively. Red, orange, cyan and black colors indicate the interacting amino acids from ACE2, identical residues between SARS-CoV and SARS-CoV-2, different residues with partially conserved contacts, and different residues with different contacts, respectively. Ridge: region between TR1 and TR2. UCR: Upper core region. A Leu to Phe486 mutation (in addition to Pro to Ala475, Pro to Glu484, Lys to Thr478, Asp to Gly476 mutations and a Val483 insertion) in TR1 increases its hydrophobicity and the order of its structure, while mutations in the ridge region of Val to Lys417 (which makes electrostatic interactions), Asn to Gln493 (more hydrogen bonding), Asp to Ser494 and Lys to Leu452 (which increase the hydrophobicity of the shallow pit) give SARS-CoV-2 an advantage to bind mainly through these regions. Arg426, Thr433 and Tyr484 in SARS-CoV (instead of Asn439, Gly446 and Gln498 in SARS-CoV-2) produce more electrostatic, hydrogen bonding and stacking interactions, respectively, which give SARS-CoVan advantage to bind mainly through TR2. The use of different regions for binding may explain the differences in affinity and pathogenesis between SARS-CoV and SARS-CoV-2. (**C**) shows the five critical binding residues that are shown in Table 2. (**D**) shows the interacting residues from ACE2 with these critical residues. Three-dimensional models were created using Discovery Studio (version 2.5.5, Biovia, San Diego, CA, USA).

Several naturally selected RBM mutations occur near the virus-binding hot regions, and these residues largely determine the host range of SARS-CoV and binding affinity. Furthermore, specific amino acids have been determined at the 442,472, 479, 480 and 487 positions of SARS-CoV (which correspond to the 455, 486, 493, 494 and 501 positions in SARS-CoV-2) that enhance viral binding to hACE2. Importantly, when all these five hACE2-favoring residues have been combined into one RBD, this RBD binds to hACE2 with super affinity and the corresponding spike protein mediates viral entry into human cells with very high efficiency [1]. Table 2 shows the changes at these five critical positions for SARS-CoV and SARS-CoV-2. Studies have highlighted positions 3 and 5 (namely Q493 and N501 in SARS-CoV-2) as the most critical residues in the binding process. Figure S2 shows the contacting ACE2 residues with RBM region, the interacting residues in the RBM region of SARS-CoV and SARS-CoV-2 are shown in Figure 11A,B, respectively. Figure 11C shows the residues that are reported in Table 2 in black (the RBD is shown in violet while the RBM is shown in pink). The contacts between these residues in SARS-CoV-2 and the corresponding ACE2 that have been observed in X-ray [2] structure are shown in Figure 11D, which shows stacking between Asn501 of the RMB and Tyr41 of ACE2, hydrogen bonding between Gln493 of the RBM with Glu35 of ACE2, stacking between Leu455 of the RBM and His34 of ACE2, and stacking between Phe486 of the RBM with Tyr83 of ACE2.

It has been determined that at position 1, phenylalanine shows more affinity to ACE2 than tyrosine does in the human, while tyrosine shows more affinity to ACE2 than phenylalanine does at the same position in the civet. For position 2 in the human, phenylalanine has more affinity to ACE2 than leucine, which, in turn, has more affinity than proline. In contrast, in the civet, proline and leucine have the same affinity to ACE2, which is greater than that of phenylalanine. Position 3 shows similar affinity in the human to ACE2 when it contains either asparagine or arginine, and either of these shows much more affinity than lysine. For civet at the same position, arginine has more affinity than either lysine or asparagine. At position 4, aspartate has more affinity than glycine in the human and vice versa in civet. Finally, at position 5, threonine has much more affinity for ACE2 than serine in the human, with a lesser difference in civet [1].

**Figure 12:**
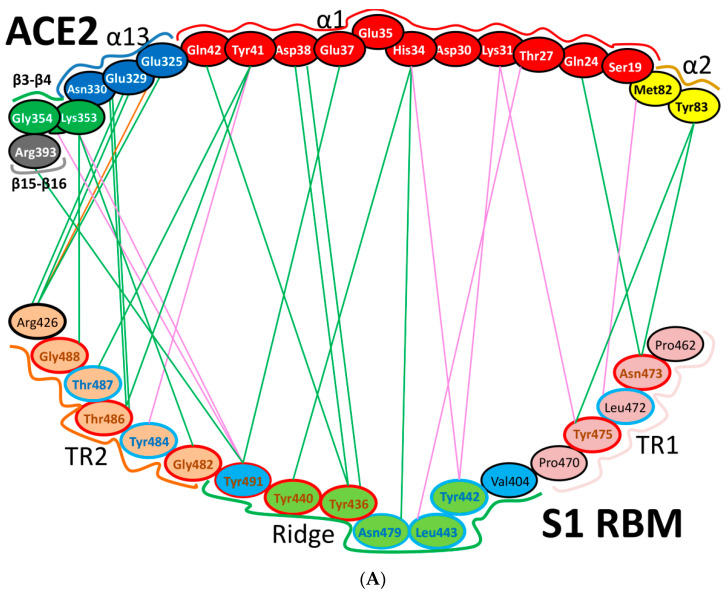

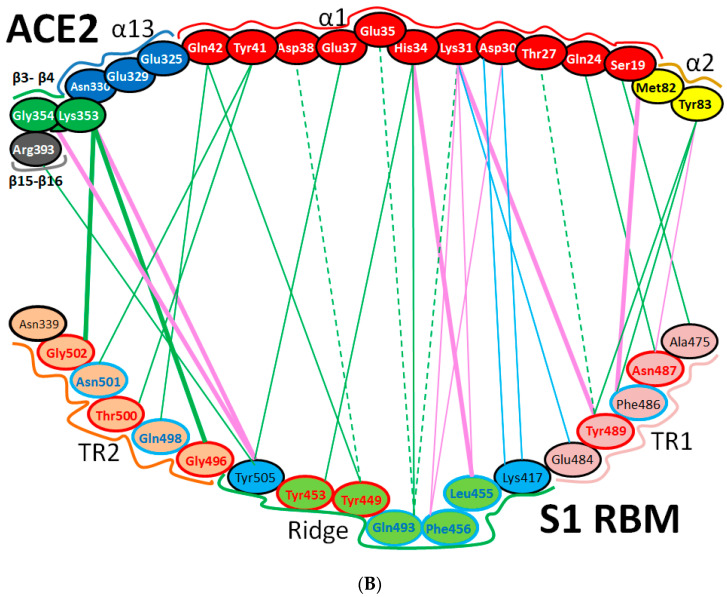
Cartoon representation of the interactions between ACE2 and the RBM of SARS-CoV and SARS-CoV-2. (**A**) SARS-CoV (based on PDB: 2AJF) and (**B**) SARS-CoV-2 (based on PDBs: 6LZG and 6M17). Hydrogen bonding interactions, hydrophobic interactions, and electrostatic interactions are shown by green, pink and orange lines, respectively. In the RBM regions, residues with red outlines are identical, while residues with cyan outlines are different amino acids with partially conserved contacts. The black outlines show different amino acids with different contacts. For interactions that are shown for SARS-CoV-2, the solid lines are attributed to interactions monitored in PDB:2AJF, while the dashed lines are attributed to those in PDB:6M17. Thick lines represent the common interactions between the two SARS-CoV-2 PDBs. “α” and “β” refer to alpha helix and beta sheet, respectively.

Further detailed comparison of the receptor-binding interface between the two coronaviruses reveals that, among the 24 residues in ACE2 that make vdw contacts with either RBD, 15 amino acids display more contacts with the SARS-CoV-2 RBD than with the SARS-CoV RBD. The SARS-CoV-2 RBD binding interface also has more residues than the SARS-CoV RBD (21 vs. 17) that directly interact with ACE2. These interacting residues form more vdw contacts (288 vs. 213) as well as more hydrogen bonds (16 vs. 11). Specifically, F486 in the RBD of SARS-CoV-2, in contrast to I472 in the SARS-CoV RBD, forms strong aromatic-aromatic interactions with hACE2 Y83 (Figure 11 and Figure 12). Similarly, E484 in the SARS-CoV-2 RBD, instead of P470 in the SARS-CoV RBD, forms ionic interactions with K31 [14] (Figure 11 and Figure 12 and Table 3). Interestingly, it has been observed that a single K353A mutation in ACE2 insufficient to abolish these interactions in SARS-CoV-2 [2].

The major electrostatic contributions are observed for the (RBMR426)/(ACE2 E329) salt bridge with SARS-CoV and (RBMK404)/(ACE2 D30) with SARS-CoV-2, as shown in (Table 3, Figure 11 and Figure 12) [104]. However, the latter exhibits a higher electrostatic interaction by 1.4 Kcal/mol. The contribution in the free-energy formation of the salt-bridges (∆G) is dominated by the pairwise acid/base interactions [104]. The total electrostatic interaction between SARS-CoV-2 and ACE2 is 3 Kcal/mol higher than that between SARS-CoV and ACE2. The contribution of a single mutation to the electrostatic binding energies is small. However, these mutations induce structural changes that increase the favorable vdw interactions in SARS-CoV-2. The total vdw contribution to the binding energy is 4 Kcal/mol, which illustrates the overall structural changes in the S protein in SARS-CoV-2. It has been found that the total binding energy in the case of SARS-CoV-2 is stronger than that in SARS-CoV, as the electrostatic and vdw interactions are greater in SARS-CoV-2 by 3 Kcal/mol and 1 Kcal/mol, respectively, than in the case of SARS-CoV [104].

In summary, SARS-CoV-2 seems to involve in more interactions with ACE2 than SARS-CoV (Figure 11 and Figure 12). There are significant differences in the TR1, middle ridge region and TR2 of the RBM in SARS-CoV-2, compared with the same RBMs parts of MERS-CoV and SARS-CoV. Thanks to these differences, SARS-CoV-2 has acquired the advantage of binding mainly through the RBM by using hydrophobic, hydrogen-bonded and electrostatic interactions in TR1 and the middle ridge. This differs from SARS-CoV, which interacts through more electrostatic links in TR2 of the RBM, together with hydrogen bonds and stacking interactions. Different types and numbers of interactions and different regions of binding between SARS-CoV-2 and SARS-CoV may explain many of their different tendencies for infection. SARS-CoV-2 and SARS-CoV both bind through TR1, middle ridge and TR2. However, SARS-COV-2 manifests more interactions (i.e., hydrophobic, hydrogen-bonded and electrostatic interactions) in TR1 and middle ridge, while SARS-CoV shows more preference for TR2 (i.e., it has more electrostatic interactions in this region than SARS-CoV-2). Furthermore, it seems that proline plays an important role in both TR1 and TR2, as it is involved in important motifs in these regions, including motifs that were well reported to affect the flexibility and disorder of the comprised regions [2,22,45,53,55,56,57].

Water solvation may play a significant role in the determination of the dynamics of the S protein in different parts, and the overall rigidities of the S protein in the two viruses may contribute to the differences between the two types. The TR1 (also referred as loop 3, L3) is markedly more rigid in the SARS-CoV-2/ACE2 adduct as compared to that of SARS-CoV [22]. Indeed, TR1 in SARS-CoV-2 possesses a more defined secondary structure (composed by small β-sheets), which is preserved along the MD simulations. Ostensibly, TR1’s length is different in the two SARS variants, being characterized by the insertion of Gly482, in SARS-CoV-2. This makes TR1 longer and more structured, enabling it to gain stabilizing interactions (namely, the mutated residues Gly485 with Cys488 and Gln474 with Gly476) in the SARS-CoV-2/ACE2 adduct. This amino acid insertion, along with other amino acidic mutations, stunningly convert an unessential part of the RBM into a strikingly effective recognition grasp for ACE2, allowing SARS-CoV-2 to stiffen, by establishing stronger interfacial interactions [22]. The presence of potent S1/S2-1 furin-like cleavage sites in SARS-CoV-2 and certain glycosylation tendencies may have critical roles in the tropism and pathogenesis of COVID-19. It is also possible, but thus far unproven, that other mutations that are distal from the RBM may contribute to such differences, and affect the binding.

## 9. S Protein and ACE2 Differences Determine the Risk of Being an Intermediate Host

Amino acids in RBM region of S protein and their contacts are critical for the binding of viruses in human and other animal reservoirs, and they can be used to assess potential intermediate hosts. For example, it was mentioned in Section 9 that a single K353A mutation in ACE2 was sufficient to abolish the interactions of ACE2 with SARS-CoV-2. Interestingly, most mammals (human, civet, bat, pig, ferret, cat, orangutan and monkey) have lysine at this position, but some mammals, such as mouse and rat, have histidine instead of lysine at this position. This difference requires assessment of the effect on their risk of being intermediate hosts [1].

On the basis of structural information regarding the complex of hACE2 and the RBD of SARS-CoV-2, the affinity to the S protein of the 20 key residues in ACE2 from mammal, bird, turtle and snake has been analyzed. Several ACE2 proteins from primates, bovidae, cricetidae and cetacea have been found to maintain the majority of key residues in ACE2 that associate with the SARS-CoV-2 RBD. Simulated structures have indicated that ACE2 proteins from bovidae and cricetidae are able to associate with the SARS-CoV-2 RBD, which suggests that bovidae and cricetidae should be included in the screening of intermediate hosts for SARS-CoV-2 [47]. Moreover, it has been found that nearly half of the key residues in turtle, snake and bird are changed. The simulated structures show that several key contacts with the SARS-CoV-2 RBD in turtle and snake ACE2 are eliminated. This finding indicates that neither snakes nor turtles maybe intermediate hosts for SARS-CoV-2, which further reinforces the concept that reptiles are resistant to infection by coronaviruses. However, other studies have indicated that interaction between the key amino acids of the S protein RBD and ACE2 in turtles may lead to these animals acting as potential intermediate hosts that can transmit SARS-CoV-2 to humans [46]. It has also been previously suggested that pangolins and snakes may act as intermediate hosts.

It has also been found that most mammals, including pets (dog and cat), pangolins and Cricetidae mammals retain the most key residues in ACE2 for association with the S protein from SARS-CoV and SARS-CoV-2. The interaction interface between the ACE2 of these mammals and the SARS-CoV/SARS-CoV-2 S protein has been manipulated through homology modeling. These studies identified a closer contact between Asn82 in ACE2 and the SARS-CoV-2 S protein than that between M82 in human ACE2 and the SARS-CoV-2 S protein. These findings provide important insights into the host range of SARS-CoV-2 and a new strategy to design an optimized ACE2 for SARS-CoV-2 infection [34,47].

## 10. Conclusions

SARS-CoV-2 has become known across the world in the last few months. It shows a pathogenesis spectrum and affinity to host receptors that are different from those of SARS-CoV and MERS-CoV. SARS-CoV-2 shares high similarity with SARS-CoV, nevertheless, small differences between them might be used to explain differences in behavior. Noticeably, the RBM of the SARS-CoV-2 S protein shows significant differences from those of SASR-CoV and MERS-CoV. For instance, the mutation of Leu to Phe486 (in addition to Pro to Ala475, Pro to Glu484, Lys to Thr478, Asp to Gly476 and a Val483 insertion) in the TR1 region makes it a more hydrophobic and ordered structure than that present in the other coronaviruses. The changes in the ridge region of Val to Lys417 (which increases the electrostatic interactions), Asn to Gln493 (which increases the number of hydrogen bonds), Asp to Ser494 and Lys to Leu452 (which make the shallow pit more hydrophobic) may give SARS-CoV-2 an advantage to bind mainly through these regions. In comparison, the presence of Arg426, Thr433 and Tyr484 in SARS-CoV, instead of Asn439, Gly446 and Gln498 in SARS-CoV-2, increases the number of electrostatic interactions, hydrogen bonds and stacking interactions in the TR2 region, respectively, and these alterations give SARS-CoV an advantage to bind mainly through TR2. Different types and numbers of interactions and different regions between SARS-CoV and SARS-CoV-2 may explain many of their different tendencies for infecting different hosts and for binding particular host receptors in specific ways. Moreover, it seems that proline plays a central role in TR1 and TR2 structure and interactions (through the appearance or disappearance of important motifs in these regions, and its role in the formation of different ordered structures). Solvation might play significant roles in the determination of the dynamics of the protein in different parts. The overall rigidity, flexibility and disorder of the different regions of S protein in the two viruses may additionally contribute to such differences. The presence of additional potent furin cleavage site (S1/S2-1) in SARS-CoV-2 and different glycosylation propensities may play critical roles in the tropism, pathogenesis and binding of the protein with drugs and antibodies. More studies are needed to investigate the effects of combinations of these factors, and computational studies may provide information to elucidate many of these concerns.

## Figures and Tables

**Table 1:** Different proteins of coronaviruses and their functions [19,21,23].

Protein	Function
nsp1, nsp3	Inhibition of IFN signaling and blocking of host’s innate immune response by promotion of cellular degradation and blockage of translation of host’s RNA.
nsp2	Binding to prohibition protein.
nsp3	Papain-like protease domain, it promotes cytokine expression and cleavage of viral polyprotein.
nsp4, nsp6	Contribution to structure of DNA methylation valleys (DMVs) as transmembrane scaffold protein (DMVs formation).
nsps5	3C-like protease domain.
nsp7, nsp8	Processivity clamp for RNA polymerase by arms hexadecameric complex; nsp 8 has adenylyltransferase activity.
nsp9	RNA binding protein phosphatase.
nsps12	RNA-dependent RNA polymerase.
nsp10, nsp16, nsp14	Stimulation of ExoN and 2-O-MT activity.
nsp13	RNA helicase, 5′ triphosphatase.
nsp14	Proofreading of viral genome.
S protein	Forms homotrimers that protrude in the viral surface and facilitate binding of envelope viruses to host cells by attraction with ACE2 that is expressed in lower respiratory tract cells. This glycoprotein is cleaved by the host cell furin-like protease into two subunits, S1 and S2. S1 is responsible for the determination of the host virus range and cellular tropism with the receptor binding domain make-up, while S2 mediates virus fusion in transmitting host cells.
N protein	Structural component localizes in the endoplasmic reticulum-Golgi region that structurally is bound to the nucleic acid material of the virus. The protein is bound to RNA, so the protein is involved in processes related to the viral genome, the viral replication cycle, and the cellular response of host cells to viral infections. N protein is also heavily phosphorylated and it is suggested that its presence leads to structural changes that enhance the affinity for viral RNA.
M protein	The most tightly structured protein, it plays a role in the determination of the shape of the virus envelope. This protein can bind to all other structural proteins. Binding with M protein helps to stabilize nucleocapsids or N proteins and promotes completion of viral assembly by stabilizing N protein-RNA complex, inside the internal virion.
E protein	The smallest of the major structural proteins. It plays a role in the production and maturation of this virus.

**Table 2:** The variants of RBM at five critical positions in SARS-CoV (442, 472, 479, 480 and 487).

Virus	Position 1	Position 2	Position 3	Position 4	Position 5
SARS-CoV	F > Y, Y, S	F > L > P, L = P, F	N, R > K = N, N	D > G, G > D, D	T >> S, S, N
SARS-CoV-2	L	F	Q	S	N

Red color is for human, cyan for civet and violet for bat. The corresponding positions in SARS-CoV-2 for human are Leu455, Phe486, Qln493, Ser494 and Asn501. The effects on affinity with ACE2 of different mutations at any position for any reservoir are indicated by symbols like “>” (greater than), “>>” (much greater than), and “=” (equal to).

**Table 3:** Interactions between amino acids in the RBDs of SARS-CoV-2 and SARS-CoV with ACE2.

Region	Number	Amino Acid	Amino Acid ACE2	Interactions	Details
TR1	1	A475	S19	Strong polar contacts	H: A475(O)-S19(HG): 3.2 (6LZG)
		P462	-	No interactions	
	2	N487	Q24	Strong polar contacts	H: N487(ND2)-Q24(HE21): 2.2 (6LZG) H: N487(HD22)-Y83(OH): 2.3 (6LZG)
		N473	Q24,Y83	Strong polar contacts	H: N473(ND2)-Q24(OE1): 2.5 (2AJF) H: N473(ND2)- Y83(HH): 2.5 (2AJF)
	3	T486	N330	Hydrogen bonding	H: T486(O)-N330(HD22): 2.3 (2AJF) H: T486(OG1)-Y41(HH): 2.6 (2AJF) H: T486(O)-N330(HD22): 2.3 (2AJF)
		F486	Y83, M82	Stacking and hydrophobic interactions	ST (Pi-Pi): F486-Y83: 5.2 (6LZG) D (Pi-alkyl): F486-M82: 4.6 (6LZG, 6M17)
	4	Y489	K31	Hydrophobic and hydrogen interactions	ST (Pi-alkyl): Y489-K31: 4.8 (6LZG, 6M17) H: Y489(HH)-Y83(OH): 3.0 (6LZG) H: Y489(HH)-T27(O): 1.7 (6M17)
		Y475	K31	Hydrophobic and hydrogen interactions	ST (Pi-alkyl): Y475- K31: 5.2 (2AJF) H: Y475(HH)-Y83(OH): 2.8 (2AJF)
	5	E484	K31	Strong polar contacts	S: E484(OE2)-K31(NZ): 4.2 (6LZG)
		P470	-	No interactions	-
Ridge and upper core region	6	K417	D30	Electrostatic interactions (Salt bridge)	HS: K417(HZ1)-D30(OD1): 2.1 (6LZG) HS: K417(HZ3)-D30(OD2): 3.0 (6LZG)
		V404	-	No interactions	-
	7	L455	K31, H34	Hydrophobic interactions	D (Pi-alkyl): L455-H34: 3.5 (6LZG, 6M17) D: L455(CD2)-K31(CA): 4.3 (6LZG, 6M17)
		Y442	K31, H34	-	ST: (Pi-Pi): Y442-H34: 6.0 (2AJF) D:Y442(CE1)-K31(CA): 4.1 (2AJF)
	8	F456	D30,K31	Stacking	ST: (Pi-alkyl): F456-K31: 4.5(6M17) ST: (Pi-alkyl): F456-D30: 4.4 (6M17)
		L443	T27	Hydrophobic interactions	D: L443(CD2)-T27(CG2): 4.2 (2AJF)
	9	Q493	K31, H34, E35	Hydrogen bonding	H: Q493(HE22)-E35(OE2): 1.9 (6M17) H: Q493(HE21)-H34(O): 2.9 (6LZG) H: Q493(OE1)-K31(HZ2/HZ3): 3.0 (6M17)
		N479	H34	-	H: N479(HD21)-H34(ND1): 3.1 (2AJF)
	10	Y449	D38, Q42	Hydrogen bonding	H: Y449(HH)-D38(OD1): 2.8 ((6M17) H: Y449(OH)-Q42(OE1): 2.5 (6LZG)
		Y436	D38	Hydrogen bonding	H: Y436(HH)-D38(OD1): 2.8 (2AJF) H: Y436(HH)-D38(OD2): 3.4 (2AJF) H: Y436(HH)-Q42(OE1): 3.4 (2AJF)
	11	Y453	H34	Polar contacts	H: Y453(HH)-H34(NE2): 3.2 (6M17)
		Y440	H34	Polar contacts	H: Y440(HH)-H34(NE2): 3.3 (2AJF)
	12	Y505	-	-	H: Y505(HH)-E37(OE1): 2.6 (6LZG, 6M17) H: Y505(OH)-R393(HH22): 3.4 (6LZG, 6M17) ST: (Pi-amid): Y505-K353/Gly354: 4.0 (6LZG, 6M17) ST (Pi-alkyl):Y505-K353: 4.6 (6LZG, 6M17)
		Y491	-	-	H: Y491(HH)-E37(OE1): 2.4 (2AJF) H: Y491(OH)-R393(HH22): 3.4 (2AJF) ST: (Pi-amid): Y491-K353/Gly354: 4.1 (2AJF) ST: (Pi-alkyl): Y491-K353: 4.3 (2AJF)
TR2	13	G496	K353 *	Hydrogen bonding	H: G496(O)-K353(HZ2/HZ3): 2.3 (6LZG, 6M17)
		G482	K353 *	Hydrogen bonding	H: G482(O)-K353(HZ2/HZ3): 3.9 (2AJF)
	14	Q498	Q48	Hydrogen bonding	H: Q498(HE22)-Q42(OE1): 2.2 (6LZG)
		Y484	Y41	Stacking	ST (Pi-Pi): Y484-Y41: 5.3 (2AJF)
	15	T500	Y41	Hydrogen bonding	H: T500(OG1)-Y41(HH): 2.8 (6LZG, 6M17)
		T486	N330	Hydrogen bonding	H: T486(O)-N330(HD22): 2.3 (2AJF) H: T486(OG1)-Y41(HH): 2.6 (2AJF) H: T486(O)-N330(HD22): 2.3 (2AJF)
	16	N501	Y41	Hydrogen bonding	H: N501(HD21)- Y41(OH): 2.3 (6LZG)
		T487	Y41	Hydrogen bonding	H: T487(HG1)- Y41(HH): 4.0 (2AJF)
	17	G502	K353 *	Hydrogen bonding	H: G502(HN)-K353(O): 1.7 (6LZG, 6M17)
		G488	K353 *	Hydrogen bonding	H: G488(HN)-K353(O): 1.5 (2AJF)
	18	N439	-	No interactions	-
		R426	E329, Q325	-	S: R426(NH2)-E329(OE2): 3.0 (2AJF) H: R426(HH21)-Q325(OE1): 3.5 (2AJF) H: R426(HH12)-E329(OE2): 3.1 (2AJF) H: R426(HH22)-E329(OE2): 2.0 (2AJF)

H: hydrogen bonding. S: Electrostatic interaction. HS: hydrogen and electrostatic interaction. D: hydrophobic interaction. ST: stacking interaction. Details include bond type: RBD amino acids (interacting atom)-ACE2 (interacting amino acids): distance in angstrom and (PDB IDs). Measured distance is based on PDB ID: 2AJF for SARS-CoV, and based on PDB IDs: 6LZG and 6M17 (if the contact is not found in 6LZG) for SARS-CoV-2. Orange colour indicates identical residues, cyan indicates different residues with partial conserved interactions, while grey indicates different residues between SARS-CoV-2 and SARS-CoV that have different contacts. For each amino acid, the upper cell corresponds to SARS-CoV-2, while the lower one corresponds to SARS-CoV. Residues in red font are non-conserved ACE2 amino acids in different mammals [1]. * A single K353A mutation was sufficient to abolish the polar contacts mediated by hydrophilic residues which dominate the virus-receptor engagement [2].

## Supplementary Materials

The following are available online at https://www.mdpi.com/2073-4409/9/12/2638/s1, Figure S1. Structural alignment of TR2 for both SARS-CoV (green) and SARS-CoV-2 (red); the annotated amino acids represent those that differ between the two viruses; Figure S2. The main contacting residues from ACE2 with RBM region.
