# Supplementary material for: Comprehensive Structural and Molecular Comparison of Spike Proteins of SARS-CoV-2, SARS-CoV and MERS-CoV, and Their Interactions with ACE2
Source: Cells. 2020 Dec 8;9(12):2638. doi: 10.3390/cells9122638 (PMC7763676; doi:10.3390/cells9122638)
Supplement: Supplementary file 1 [file cells-09-02638-s001.pdf]

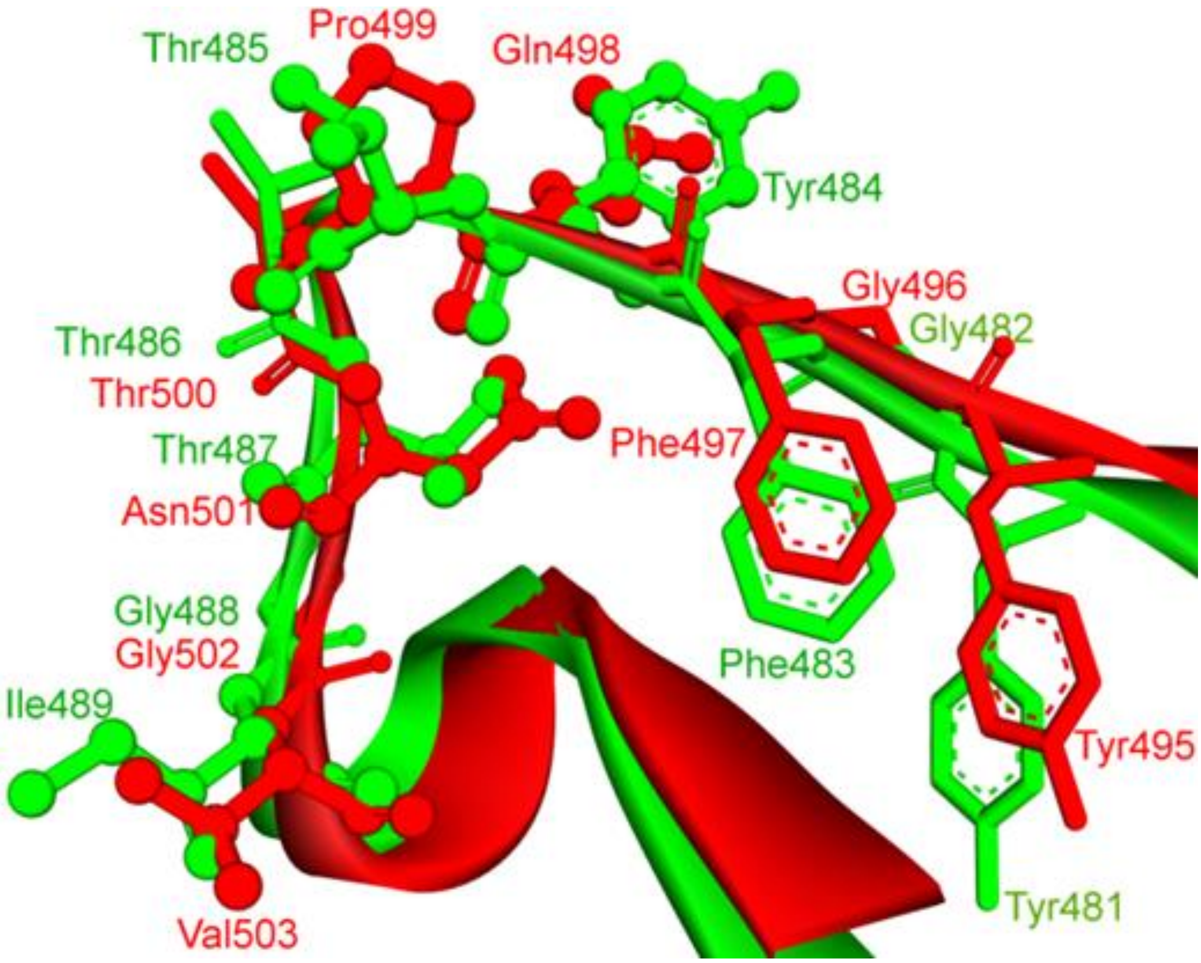

Figure S1. Structural alignment of TR2 for both SARS-CoV (green) and SARS-CoV-2 (red); the annotated amino acids represent those that differ between the two viruses. Created using Discovery Studio (version 2.5.5, Biovia, USA).

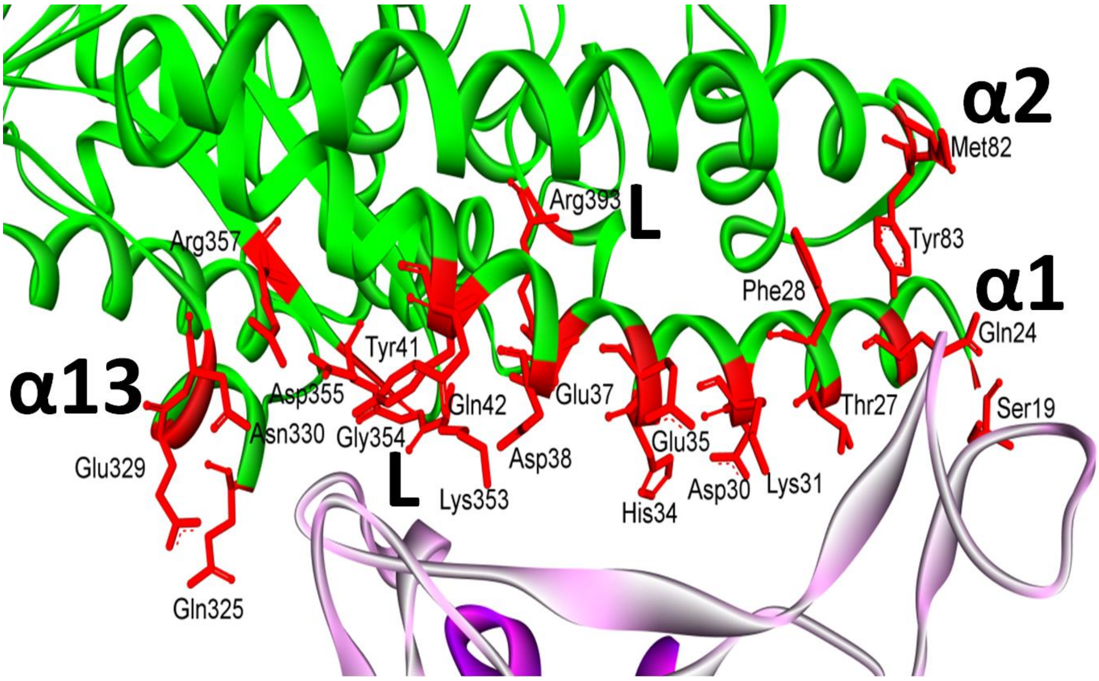

Figure S2. The main contacting residues from ACE2 with RBM region. Created using Discovery Studio (version 2.5.5, Biovia, USA).
